# Early immune factors associated with the development of post-acute sequelae of SARS-CoV-2 infection in hospitalized and non-hospitalized individuals

**DOI:** 10.3389/fimmu.2024.1348041

**Published:** 2024-01-22

**Authors:** Jacqueline M. Leung, Michelle J. Wu, Pouya Kheradpour, Chen Chen, Katherine A. Drake, Gary Tong, Vanessa K. Ridaura, Howard C. Zisser, William A. Conrad, Natalia Hudson, Jared Allen, Christopher Welberry, Celine Parsy-Kowalska, Isabel Macdonald, Victor F. Tapson, James N. Moy, Christopher R. deFilippi, Ivan O. Rosas, Mujeeb Basit, Jerry A. Krishnan, Sairam Parthasarathy, Bellur S. Prabhakar, Mirella Salvatore, Charles C. Kim

**Affiliations:** ^1^ Verily Life Sciences, South San Francisco, CA, United States; ^2^ Providence Little Company of Mary Medical Center Torrance, Torrance, CA, United States; ^3^ Oncimmune Limited, Nottingham, United Kingdom; ^4^ Department of Medicine, Cedars-Sinai Medical Center, Los Angeles, CA, United States; ^5^ Department of Internal Medicine, Rush University Medical Center, Chicago, IL, United States; ^6^ Inova Schar Heart and Vascular, Falls Church, VA, United States; ^7^ Department of Medicine, Baylor College of Medicine, Houston, TX, United States; ^8^ Department of Internal Medicine, University of Texas Southwestern Medical Center, Dallas, TX, United States; ^9^ Breathe Chicago Center, University of Illinois Chicago, Chicago, IL, United States; ^10^ Division of Pulmonary, Allergy, Critical Care & Sleep Medicine, University of Arizona, Tucson, AZ, United States; ^11^ Department of Microbiology and Immunology, University of Illinois - College of Medicine, Chicago, IL, United States; ^12^ Department of Medicine and Department of Population Health Sciences, Weill Cornell Medicine, New York, NY, United States

**Keywords:** COVID-19, PASC, long COVID, autoantibody, double-negative B cells

## Abstract

**Background:**

Infection by severe acute respiratory syndrome coronavirus 2 (SARS-CoV-2) can lead to post-acute sequelae of SARS-CoV-2 (PASC) that can persist for weeks to years following initial viral infection. Clinical manifestations of PASC are heterogeneous and often involve multiple organs. While many hypotheses have been made on the mechanisms of PASC and its associated symptoms, the acute biological drivers of PASC are still unknown.

**Methods:**

We enrolled 494 patients with COVID-19 at their initial presentation to a hospital or clinic and followed them longitudinally to determine their development of PASC. From 341 patients, we conducted multi-omic profiling on peripheral blood samples collected shortly after study enrollment to investigate early immune signatures associated with the development of PASC.

**Results:**

During the first week of COVID-19, we observed a large number of differences in the immune profile of individuals who were hospitalized for COVID-19 compared to those individuals with COVID-19 who were not hospitalized. Differences between individuals who did or did not later develop PASC were, in comparison, more limited, but included significant differences in autoantibodies and in epigenetic and transcriptional signatures in double-negative 1 B cells, in particular.

**Conclusions:**

We found that early immune indicators of incident PASC were nuanced, with significant molecular signals manifesting predominantly in double-negative B cells, compared with the robust differences associated with hospitalization during acute COVID-19. The emerging acute differences in B cell phenotypes, especially in double-negative 1 B cells, in PASC patients highlight a potentially important role of these cells in the development of PASC.

## Introduction

Since 2019, the Coronavirus Disease 2019 (COVID-19) pandemic, caused by infection with severe acute respiratory syndrome coronavirus 2 (SARS-CoV-2), has caused significant morbidity and mortality around the world. Although rates of hospitalizations and deaths from COVID-19 have declined in recent years (1), COVID-19 remains a global public health challenge and was ranked as the fourth leading cause of death in the United States in 2022 (2). COVID-19 is characterized by a spectrum of illnesses ranging from asymptomatic infection to severe disease and mortality. While the majority of individuals recover from COVID-19, a subset of SARS-CoV-2-infected individuals experience persistent (or emerging) symptoms that can last for weeks to years following initial infection (3), a condition known as post-acute sequelae of SARS-CoV-2 infection (PASC) or long COVID. Individuals with PASC experience a wide range of symptoms affecting multiple organ systems, including symptoms such as loss of taste or smell, post-exertional malaise, fatigue, brain fog, gastrointestinal symptoms, chronic cough, and chest pain, among others (4). The biological driver(s) of the diverse manifestations of PASC are currently unknown (5), and it is still unclear why some individuals develop PASC while others do not. Emerging evidence suggests that PASC development is associated with long lasting dysregulation of the immune response that may be a consequence from various factors including excessive inflammatory responses due to viral activation, viral reservoirs persisting in infected tissues, gut dysbiosis, microvascular dysfunction, and autoimmunity to self-antigens (6, 7).

A number of studies have evaluated the immune response during acute COVID-19 and between individuals with and without established PASC (8–15). However, the early immune response during acute SARS-CoV-2 infection in individuals who eventually do and do not develop PASC remains relatively understudied. In this study, we collected peripheral blood samples from COVID-19 patients during their initial presentation to an ambulatory clinic or hospital in the early stages of the pandemic (May 2020 to June 2021) and followed them longitudinally to determine their development of PASC. We conducted multi-omic assays on samples collected at hospital and clinic presentation, with the aim of uncovering early immune mechanisms that differentiated individuals on different trajectories of PASC.

## Materials and methods

### Study design

The Predictors of Severe COVID-19 Outcomes (PRESCO) study (Trial Registration Number: NCT04388813) was a multi-center, prospective, cohort study aimed at identifying molecular and clinical features associated with the progression to severe COVID-19. Adults age 18 years and older with a confirmed, positive test for SARS-CoV-2 infection (via reverse transcription-polymerase chain reaction (RT-PCR) or antigen testing), who received care at one of eight sites across the United States (Baylor College of Medicine, Cedars-Sinai Medical Center, Inova Health Care Services, Rush University Medical Center, The University of Arizona, University of Illinois Chicago, University of Texas Southwestern Medical Center, and Weill Cornell Medical College) between May 2020 and June 2021 were invited to participate. Individuals who were pregnant were excluded from the study. Participants were followed for three months after enrollment. Enrollment for the PRESCO study was completed before the SARS-CoV-2 delta variant emerged as the predominant variant in the United States during the summer of 2021 and before the availability of the COVID-19 treatments, nirmatrelvir, ritonavir, and molnupiravir. The PRESCO study included up to five study visits: (1) at enrollment during a participants’ initial presentation to an ambulatory clinic or hospital, and, in people who were hospitalized, (2) two days after hospitalization, (3) the day of admission to an intensive care unit (if this occurred), (4) the day of hospital discharge, and, for all participants, (5) a follow-up visit three months after enrollment. Participants were followed through visit 5 or study exit for other reasons (e.g., death or lost to follow-up), whichever occurred first.

As the COVID-19 pandemic evolved, information on PASC and any remaining symptoms at the 3-month follow-up visit were collected. Participants who developed PASC (termed the “PASC” group) and participants who did not develop PASC (termed the “non-PASC” group) were grouped based on the definition of PASC from the Center for Disease Control and Prevention (16). Specifically, during the 3-month follow-up visit, participants were asked about the duration, in weeks, that it took for them, since their last study visit, to return to their usual state of health. Individuals with PASC were then defined as those individuals who did not recover to their usual state of health for four or more weeks since the start of COVID-19, which was determined by the earliest of several non-self-reported dates, including enrollment in the PRESCO study, first laboratory-confirmed positive SARS-CoV-2 test, date of hospital presentation, and hospitalization date (17).

Approval to conduct the PRESCO study was obtained by a central Western Institutional Review Board (IRB Protocol number: 20201016) and from each of the eight sites that enrolled participants. All participants or their legally authorized representatives provided written informed consent before any study-related procedures began. See the **Supplementary Material** for more details.

### Multi-omic analysis

All comparisons were conducted on blood samples collected at either hospital or clinic presentation or two days after hospitalization for hospitalized patients. Given the close proximity in the timing of these sample collections, samples from these two visits were analyzed together for all downstream analyses. All comparisons were either between hospitalized and non-hospitalized participants or PASC and non-PASC participants.

Verily’s Immune Profiler platform was used to conduct multi-omic analyses of collected blood samples. Briefly, 25 immune cell subsets, including a bulk peripheral blood mononuclear cell (PBMC) sample, 5 myeloid cell subsets, 7 B cell subsets, 10 T cell subsets, and 2 natural killer (NK) cell subsets, were isolated from approximately 10 million cryopreserved PBMCs per participant. The bulk PBMC subset was used for quality control measures only and was not analyzed further in the multi-omic comparisons. Assay for transposase-accessible chromatin using sequencing (ATAC-seq) and RNA sequencing (RNA-seq) were performed for all of the 25 subsets, and targeted protein estimation by sequencing (TaPE-seq) (18) was performed for the 12 immune cell subsets within the T and NK panel (**Supplementary Table 1**). Flow cytometry, ATAC-seq, RNA-seq, and TaPE-seq were performed as previously described (19).

### Quantification of plasma cytokines

From plasma samples, 47 cytokines (EGF, Eotaxin, FGF-2, Flt-3 ligand, Fractalkine, G-CSF, GM-CSF, GROα, IFNα2, IFNγ, IL-1α, IL-1β, IL-1ra, IL-2, IL-3, IL-4, IL-5, IL-6, IL-7, IL-8, IL-9, IL-10, IL-12p40, IL-12p70, IL-13, IL-15, IL-17A, IL-17E/IL-25, IL-17F, IL-18, IL-22, IL-27, IP-10, LTA [TNFβ], MCP-1, MCP-3, M-CSF, MDC, MIG, MIP-1α, MIP-1β, PDGF-AA, PDGF-AB/BB, sCD40L, TGFα, TNF [TNFα], and VEGF-A) were quantified using the MILLIPLEX MAP Human Cytokine/Chemokine/Growth Factor Panel A on a Luminex FLEXMAP 3D instrument, according to the manufacturer’s instructions. For each measured cytokine, concentration values that fell outside of the standard curve were imputed to the nearest standard concentration. The cytokine, GM-CSF, was excluded from further analysis because 98% of its measurements were outside of the kit’s quantification range. Downstream cytokine analyses thus included a total of 46 cytokines. Additionally, individual cytokine measurements that did not have either a) bead counts ≥ 35 and technical coefficients of variation (CV) ≤ 30%, or b) bead counts ≥ 20 and technical CV ≤ 15%, were excluded from further analysis.

### Quantification of autoantibodies and antibodies against viral antigens

Multiplexed bead-based arrays were assembled with a total of 744 antigens: 441 human proteins indicated in immune responses (and including 3 Ig controls), 114 viral proteins that included differing recombinant versions of proteins of SARS-CoV-2 as well as other viruses (MERS-CoV, SARS-CoV-1, SARS-CoV-2, HCoV-HKU1, HCoV-229E, HCoV-NL63, HCoV-OC43, Influenza A, and Influenza B), and 192 viral peptides, of which 178 originated from SARS-CoV-2 sequences and 14 were from other viruses. A full list of the human and viral proteins used in this study can be found in **Supplementary Table 2**, **Supplementary Table 3**, respectively, and is summarized in **Supplementary Table 4**. A full list of the viral peptides used can be found in **Supplementary Table 5** and is summarized in **Supplementary Table 6**. See the Methods section in the **Supplementary Material** for more details.

Assay methodology for autoantibody and viral antibody detection has been described previously (20). Briefly, beads were analyzed on a FLEXMAP 3D instrument for fluorescent signal readout, as measured by median fluorescence intensity (MFI). Measurements were excluded when there were low numbers of bead events (< 10 beads) counted per bead region. Median inter- and intra-plate CV were calculated by measuring three reference samples: one COVID-19 positive, one Systemic Lupus Erythematosus (SLE) positive, and one SLE and COVID-19 negative.

Several fixed control criteria were defined to enforce high data quality. A data completeness threshold was set at > 98% of available instrument data. Additionally, bead count statistics were controlled to disallow more than 10% drop-outs. This criterion prevented the use of MFI values for plates, samples, and antigens with insufficient bead counts. The lower MFI range was monitored via median MFI of BSA-coupled beads and was set to be below 500. Upper median MFI range of the IgG-coupled beads was set to > 20,000. The antigen panel was divided into 4 bead-based arrays for ease of processing of up to 230 bead regions. Assays contained control reference samples as well as sample-antigen pairs measured in triplicate in each plate. This allowed for control of inter- and intra-plate variance, which were both set to < 30%. Additionally, for proteins raised in *E. coli*, background reactivity of sera to *E. coli* proteins was monitored. See the **Supplementary Material** for more details.

### Differential analysis

Linear modeling methods were used for univariate differential analysis as previously described (19). Briefly, for these analyses, each molecular feature was regressed on the outcome group and appropriate clinical and technical covariates. For count-based data such as ATAC-seq, RNA-seq, and TaPE-seq, the voom-limma method was used (21). For non-count-based data such as cell subset frequencies, autoantibody/antibody concentrations, and cytokine levels, differential analysis was performed by fitting generalized linear models (GLMs). Where appropriate, data were transformed (e.g., log transformation) prior to fitting the GLMs.

Our models adjusted for a number of covariates, including demographic and clinical variables and assay-specific variables. The full model in the PASC versus non-PASC comparisons included covariates for age, sex, race, tobacco use, WHO score, and the time from COVID-19 start to sample collection. In comparing hospitalized and non-hospitalized participants, WHO score was removed as a covariate given its association with hospitalization. Association analyses with multi-omic data also adjusted for variables associated with sample quality, including neutrophil frequency (as a measure of neutrophil contamination during the isolation of PBMCs), cell viability, and the recovered subset cell counts.

Following linear modeling, the Benjamini-Hochberg procedure was used to correct for multiple hypothesis testing within each molecular data type. For the multi-omic data, comparisons were conducted per immune cell subset, and the resulting p-values across all tests within a cell subset were corrected. Significance was assessed at a false discovery rate (FDR) of 0.1, unless otherwise noted.

### Pathway analysis

Gene sets from the Molecular Signatures Database (MSigDB) were used for pathway analysis through two independent methods. First, gene set enrichment of significant differential genes were tested using hypergeometric tests. Second, Gene Set Enrichment Analysis (GSEA) (22) was conducted using effect estimates from the univariate differential analysis, which enabled the identification of gene sets where the individual genes may not be significantly differentially expressed, but are nonetheless coordinated in their association with hospitalization or PASC development.

## Results

### Overview of the PRESCO cohort and molecular data generation

A total of 494 participants with COVID-19 were enrolled in the PRESCO study, of which 354 participants had follow-up symptom surveys collected approximately 3 months after the start of COVID-19 that could inform on their development of PASC. Demographic and clinical characteristics associated with PASC in this cohort were previously described (17). Briefly, in the PRESCO cohort, participants with PASC were significantly older in age, had greater proportions of tobacco use and obesity, and had a greater proportion of Non-Hispanic White people than non-PASC participants (17). The PASC group also had more severe COVID-19 based on their WHO score, had greater usage of dexamethasone and remdesivir for COVID-19, had a higher proportion of hospitalized patients, and, for those hospitalized, had a longer duration of hospitalization compared to non-PASC participants (17).

**Figure 1:**
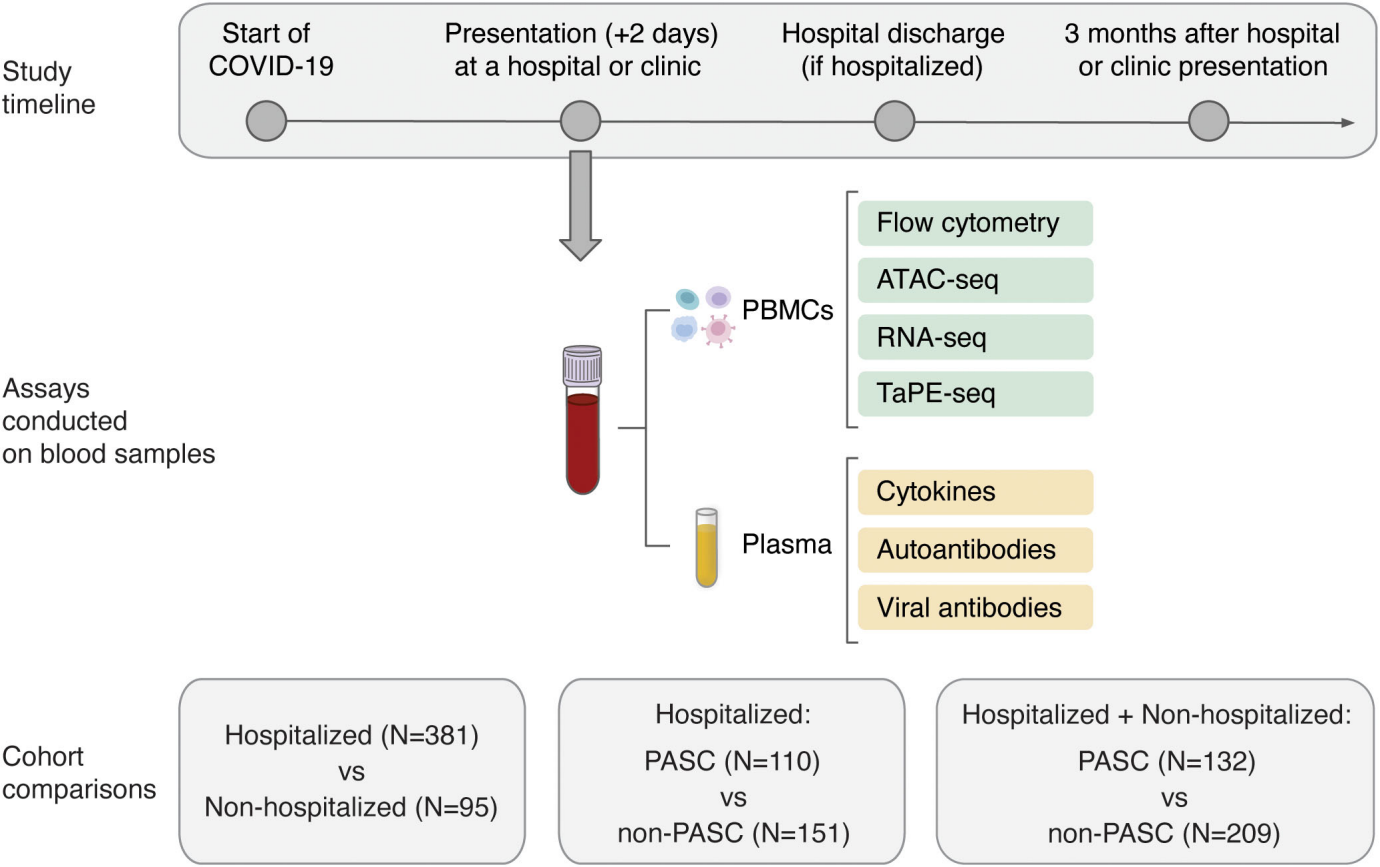
Overview of the study design and molecular assays conducted for the PRESCO study. Participants were classified based on their development of PASC over time, and samples collected during presentation at a hospital or ambulatory clinic were compared between individuals hospitalized and not hospitalized for COVID-19 and between individuals who did and did not develop PASC. The molecular assays conducted on isolated PBMCs and plasma are shown; PBMCs = peripheral blood mononuclear cells.

Peripheral blood from 476 PRESCO participants (381 hospitalized and 95 non-hospitalized participants), was available from the time of initial presentation to an ambulatory clinic or hospital for COVID-19 (**Figure 1**; **Table 1**). These samples were used to compare immune responses in hospitalized versus non-hospitalized COVID-19 patients. Of these, blood samples from 341 participants (132 PASC and 209 non-PASC) with information regarding their PASC status were also analyzed, with a focus on those that were hospitalized for COVID-19 (110 hospitalized PASC and 151 hospitalized non-PASC participants) (**Figure 1**; **Table 1**). Together, these PASC and non-PASC samples were used to investigate early molecular signatures associated with the development of PASC (**Figure 1**; **Table 1**; **Supplementary Figure 1**).

**Table 1:** Overview of sample sizes for each cohort comparison and molecular assay.

Cohort with available blood samples	Comparison groups	Total participants	Multi-omic profiling	Cytokines	Auto- antibodies
Hospitalized + non-hospitalized COVID-19 patients	Hospitalized	381	205	378	128
	Non-hospitalized	95	64	93	22
Hospitalized COVID-19 patients	PASC	110	72	109	54
	non-PASC	151	106	149	74
Hospitalized + non-hospitalized COVID-19 patients	PASC	132	85	131	60
	non-PASC	209	147	206	90

For all PASC and non-PASC participants, the blood samples used for multi-omic profiling, cytokine assessment, and autoantibody assays had a mean (standard deviation) time to collection of 3.02 (3.73) days, 3.05 (3.96) days, and 2.58 (3.72) days from the start of COVID-19, respectively. From the isolated PBMCs, 24 cell subsets, which included 5 myeloid cell subsets, 7 B cell subsets, 10 T cell subsets, and 2 NK cell subsets, were phenotyped by flow cytometry and further profiled by ATAC-seq, RNA-seq, and TaPE-seq (**Figure 1**; **Supplementary Figure 2**; **Supplementary Table 1**). Plasma samples collected during hospital or clinic presentation were also analyzed for concentrations of cytokines, autoantibodies, and antibodies against SARS-CoV-2 and other common viral antigens (**Figure 1**).

### Widespread immunological differences are observed between hospitalized and non-hospitalized COVID-19 participants during acute SARS-CoV-2 infection

PASC occurs in individuals with mild to severe COVID-19, but it is more common in patients hospitalized for more severe disease (23, 24). We hypothesized that differences in disease severity would be the strongest molecular signal during acute COVID-19, so we started with a comparison of hospitalized and non-hospitalized participants to inform our PASC comparisons. Using multi-omic profiling and assays for cytokine, autoantibody, and viral antibody detection, we compared molecular signatures between 381 hospitalized participants and 95 non-hospitalized participants at their initial presentation to a hospital or ambulatory clinic for COVID-19 (**Table 1**). Blood samples were collected at a similar timeframe since the start of COVID-19 for both hospitalized and non-hospitalized patients (mean 2.9 (SD 2.1) days for hospitalized patients versus mean 3.1 (SD 4.2) days for non-hospitalized patients) (**Supplementary Table 7**). Demographics of hospitalized and non-hospitalized patients were also summarized (**Supplementary Table 7**). Approximately 4% of all patients had a prior autoimmune disease before contracting COVID-19.

**Figure 2:**
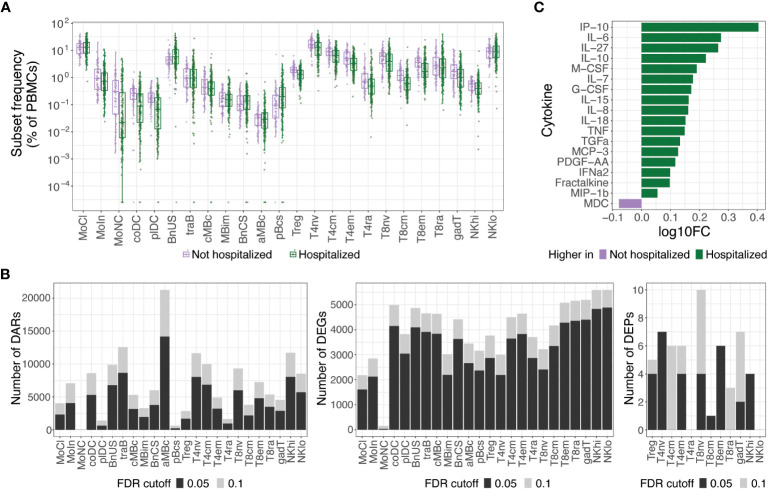
Molecular differences observed in hospitalized versus non-hospitalized participants. **(A)** Box plots of cell subset frequencies grouped by hospitalized participants and non-hospitalized participants at hospital/clinic presentation. **(B)** Identified number of significant differentially accessible regions (DARs) from ATAC-seq data, differentially expressed genes (DEGs) from RNA-seq data, and differentially expressed proteins (DEPs) from TaPE-seq data between hospitalized and non-hospitalized participants by cell subset at hospital/clinic presentation. **(C)** Cytokines and chemokines elevated in plasma of hospitalized versus non-hospitalized participants at a FDR of 0.05 as measured by Luminex assay. Comparative values are expressed as log10 fold change (log10FC); Abbreviations for immune cell subsets are defined in **Supplementary Table 1**.

At an FDR of 0.05, hospitalized participants exhibited increases in cell subset frequencies of unswitched naive B cells (BnUS) and decreases in a number of T cell subsets, including regulatory T cells (Treg), central and effector memory CD4+ T cells (T4cm, T4em), and central and effector memory CD8+ T cells (T8cm, T8em), in addition to decreased frequencies of certain myeloid cells, including conventional dendritic cells (coDC), plasmacytoid dendritic cells (plDC), and non-classical monocytes (MoNC) (**Figure 2A**). Additionally, numerous differentially accessible regions (DARs) and differentially expressed genes (DEGs) within innate and adaptive immune cells were associated with hospitalization status, highlighting diverse molecular changes early on in SARS-CoV-2 infection that can differentiate individuals on different disease severity trajectories (**Figure 2B**). At an FDR of 0.05, 46.9% of all DEGs had a proximally associated DAR in their respective cell subset, and of the differentially expressed proteins (DEPs) in the T and NK cell subsets (**Figure 2B**), nine of the 32 DEPs had coordinated changes in DARs and DEGs. These DEPs, all of which were increased in hospitalized participants, included CD127 (gene: *IL7R*) in T4em, naive CD8+ T cells, and CD56hi NK cells (NKhi), CD184 (*CXCR4*) in naive CD4+ T cells, CD38 (*CD38*) and CD366 (*HAVCR2*) in T8em, CD39 (*ENTPD1*) and CD314 (*KLRK1*) in NKhi, and CD279 (*PDCD1*) in Treg cells.

At an FDR of 0.05, numerous cytokines and chemokines in plasma were also upregulated in hospitalized participants, including key inflammatory cytokines such as IP-10, IL-6, IL-8, IL-18, TNF, and IFNα2 (**Figure 2C**). No significant differences in autoantibodies or antibodies against SARS-CoV-2 or other common viral pathogens, however, were found between hospitalized and non-hospitalized participants during acute SARS-CoV-2 infection. Given the large differences in immune status between hospitalized and non-hospitalized participants and the potential for this to be a source of variance for PASC, we further examined differences in PASC and non-PASC participants within those who were hospitalized for COVID-19.

### Alterations in double-negative B cells and interferon signaling pathways are observed in hospitalized PASC participants at initial hospital presentation for COVID-19

**Figure 3:**
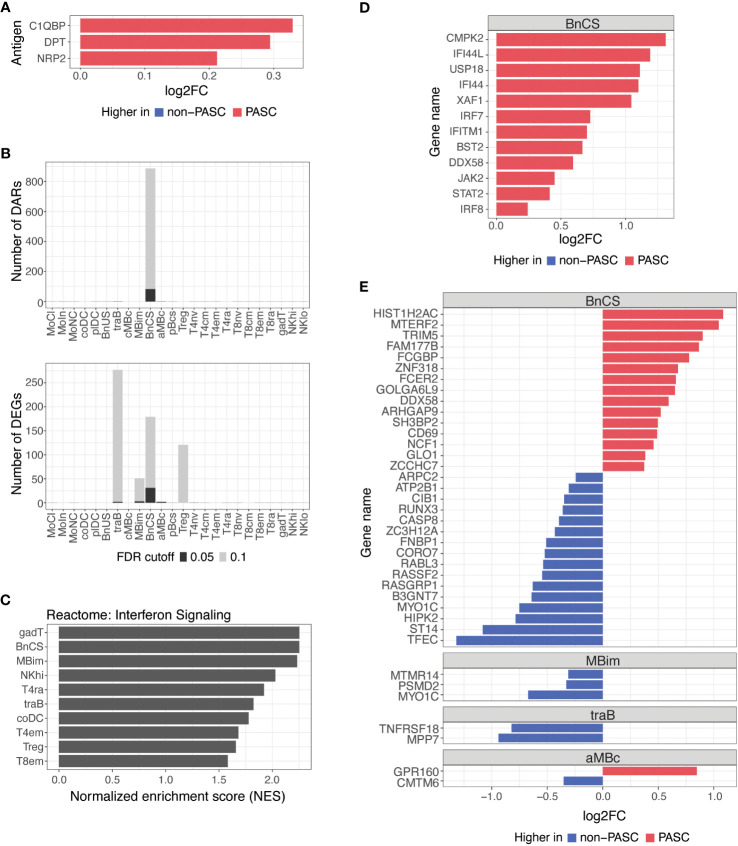
Differences in autoantibodies and in B cell epigenetic and transcriptional features in hospitalized PASC versus non-PASC participants at hospital presentation. **(A)** Log2 fold-change (log2FC) of plasma autoantibodies in hospitalized PASC versus non-PASC participants at hospital presentation at FDR of 0.1. **(B)** Identified number of significant differentially accessible regions (DARs) from ATAC-seq data and differentially expressed genes (DEGs) from RNA-seq data between hospitalized PASC and non-PASC participants by cell subset at hospital presentation. FDR cutoffs for each data type were set and graphed as FDR of 0.05 (black bar) or FDR of 0.1 (gray bar). **(C)** Pathway analysis of differentially expressed genes (DEGs) in PASC vs non-PASC participants that were hospitalized. Analysis was done for the Reactome pathway set (curated by the MsigDB collections) using a hypergeometric test of all DEGs in the 24 immune subsets. Shown are the subsets with significant enrichment of genes in the interferon signaling pathway at an FDR of 0.1. **(D)** Interferon stimulated genes that are differentially expressed in DN1 B cells at an FDR of 0.1 in PASC vs non-PASC participants that were hospitalized. **(E)** Log2FC of DEGs between hospitalized PASC and non-PASC participants at hospital presentation at an FDR of 0.05; Abbreviations for immune cell subsets are defined in **Supplementary Table 1**.

Of the hospitalized participants with molecular samples analyzed, PASC status information was available for 261 individuals, of which 110 participants eventually developed PASC and 151 participants did not develop PASC (**Table 1**). Demographics of hospitalized PASC and non-PASC patients were summarized (**Supplementary Table 8**). There were no significant differences in cell subset frequencies or plasma cytokines between hospitalized PASC versus hospitalized non-PASC participants (**Supplementary Figure 3**; **Supplementary Figure 4**). However, during acute SARS-CoV-2 infection, we observed differences in autoantibodies and in epigenetic and transcriptional signatures in B cells between PASC and non-PASC participants that were hospitalized for COVID-19 (**Figure 3**).

We found a small number of IgG autoantibodies increased in hospitalized PASC compared to hospitalized non-PASC participants at the time of hospital presentation. Hospitalized PASC participants had relative increases in three autoantibodies with reactivity to the complement protein, Complement C1q Binding Protein (C1QBP), an adipokine, Dermatopontin (DPT), and a SARS-CoV-2 entry receptor, Neuropilin-2 (NRP2) (**Figure 3A**). However, no significant differences in antibodies against SARS-CoV-2 or other common viral pathogens were observed.

B cells are the major effector cells responsible for antibody production and also contribute to other effector functions such as cytokine production and immune regulation. At an FDR of 0.05, the predominant differences between hospitalized PASC and non-PASC participants during acute SARS-CoV-2 infection were found in B cell subsets for both DARs and DEGs (**Figure 3B**). Specifically, we found that the majority of DARs identified by ATAC-seq were observed in double-negative (DN; CD27-IgD-) B cells of the DN1 subset (BnCS) (**Figure 3B**). Double-negative B cells lack CD27 expression, making them similar to naive B cells, but they also lack IgD expression, suggesting that they have undergone immunoglobulin isotype switching similar to switched memory B cells (25, 26). Recently, subsets of DN B cells (DN1, DN2, DN3, and DN4) have been categorized using various markers such as CD21, CD11c, CXCR5, T-box expressed in T cells (T-bet), and Fc Receptor Like 5 (FcRL5) (25, 26), but to date, the phenotypic markers of DN B cells have not yet been standardized across studies. We classify DN1 B cells by cell surface expression of CD27- CD21+ IgM- IgD- (BnCS; **Supplementary Table 1**) and find this cell subset to exhibit the most epigenetic differences between hospitalized PASC and non-PASC participants at initial presentation to a hospital for COVID-19.

Severe COVID-19 has been associated with both impaired and overly robust type 1 interferon responses, which may either limit anti-viral immunity or exacerbate hyperinflammation, respectively (27–30), and thereby contribute to disease progression. Dysregulation of interferon responses have been observed as far as 8 months after initial SARS-CoV-2 infection (8) and may therefore be associated with the development of PASC. By conducting pathway analysis using GSEA on differentially expressed genes, we observed an enrichment of genes involved in interferon signaling in hospitalized PASC compared to non-PASC participants, particularly in effector B and T cell subsets (**Figure 3C**). Hospitalized PASC participants exhibited increased expression of numerous interferon stimulated genes (ISGs), including *CMPK2*, *IFI44L*, *USP18*, *IFI44*, *XAF1*, *IRF7*, *IFITM1*, *BST2*, *DDX58*, *JAK2*, *STAT2*, and *IRF8*, specifically in the DN1 B cell subset, during acute SARS-CoV-2 infection (**Figure 3D**). These ISGs are involved in both positive and negative regulation of the interferon signaling pathway. Interferon regulatory factor (IRF)7 and *IRF8*, for example, bind interferon-stimulated response elements and drive ISG expression in response to type I interferons (31), whereas ISGs such as *USP18*, *IFI44*, and *IFI44L* negatively regulate the type I interferon pathway and can promote viral production (32–34). As WHO score was included as a covariate in our model, the increases observed in interferon pathways and ISGs in DN1 B cells in hospitalized PASC participants occur despite controlling for disease severity. Together, these results suggest that in the acute phase of SARS-CoV-2 infection, the regulation of interferons and their impacts on anti-viral immunity and inflammation may be influencing the development of PASC.

Thirty-eight genes were also differentially expressed between hospitalized PASC and non-PASC participants at an FDR of 0.05, all of which were found within a B cell subset (**Figure 3E**). Similar to the ATAC-seq data, most DEGs from RNA-seq were found in the DN1 B cell subset. In particular, in the DN1 subset, we observed increased expression of genes related to anti-viral immune responses, including *TRIM5* and *DDX58*. *TRIM5* promotes innate immune signaling and is a restriction factor that blocks the early stages of retrovirus infection (35). *DDX58*, which encodes RIG-I, is also an innate immune sensor that recognizes double stranded RNA viruses and drives type I interferon signaling (36). Genes related to B cell activation were also upregulated in the DN1 subset of hospitalized PASC participants, including *CD69*, an early lymphoid activation marker (37, 38), and *FCER2*, which encodes CD23 and can indicate an activated B cell state (39). Together, the DEGs upregulated in DN1 B cells of hospitalized PASC participants indicate cells that have adopted an activated state and are potentially primed for anti-viral immunity. Additionally, a number of genes downregulated in DN1 B cells of hospitalized PASC participants, are involved in the process of apoptosis via various mechanisms, including *RUNX3*, *CASP8*, *RASSF2*, and *HIPK2* (40–43), and may relate to potential dysregulation of apoptotic pathways in DN1 B cells of PASC participants during acute SARS-CoV-2 infection. A smaller number of significant gene differences were observed in IgM+ IgD- classical memory B cells, transitional B cells, and atypical memory B cells at an FDR of 0.05 (**Figure 3E**).

### Minor alterations in autoantibodies and B cell epigenetic and transcriptional signatures are observed in hospitalized and non-hospitalized PASC participants during acute SARS-CoV-2 infection

**Figure 4:**
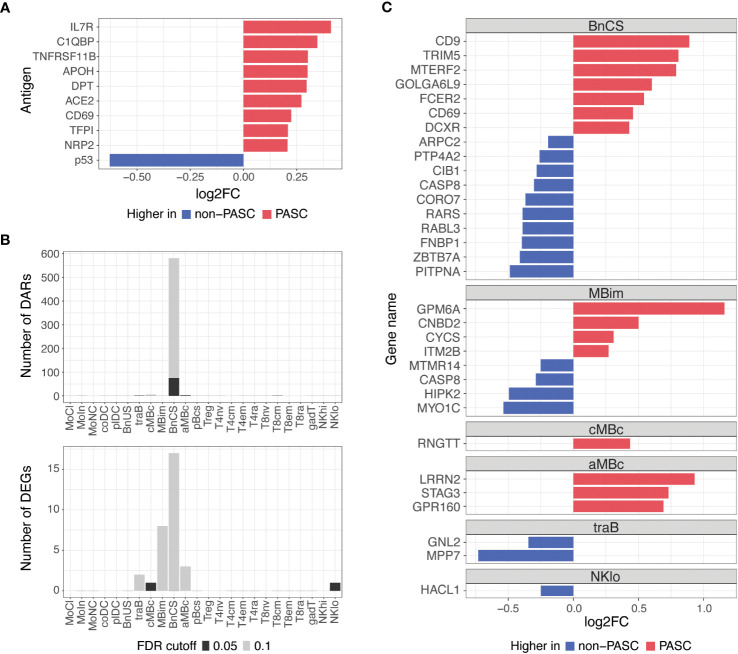
Differences in autoantibodies and in B cell epigenetic and transcriptional features in PASC versus non-PASC participants at hospital/clinic presentation. **(A)** Log2 fold-change (log2FC) of plasma autoantibodies in PASC versus non-PASC participants at hospital/clinic presentation at FDR of 0.1. **(B)** Identified number of significant differentially accessible regions (DARs) from ATAC-seq data and differentially expressed genes (DEGs) from RNA-seq data between PASC and non-PASC participants by cell subset at hospital/clinic presentation. FDR cutoffs for each data type were set and graphed as FDR of 0.05 (black bar) or FDR of 0.1 (gray bar). **(C)** Log2FC of DEGs between PASC and non-PASC participants at hospital/clinic presentation at FDR of 0.1; Abbreviations for immune cell subsets are defined in **Supplementary Table 1**.

To understand differences between individuals with and without PASC more broadly, we examined molecular signatures between PASC and non-PASC in the full cohort, which included both hospitalized and non-hospitalized participants (**Figure 1**; **Table 1**). At the time of a participants’ initial presentation to a hospital or ambulatory clinic for COVID-19, we detected fewer molecular differences between PASC and non-PASC participants in the full cohort compared to the hospitalized group only, but similar overlapping signals between the two cohorts were observed. There were no significant differences in immune cell subset frequencies or plasma cytokines between PASC and non-PASC participants during acute COVID-19. Similar to the hospitalized cohort, we observed increased autoantibodies against the three antigens, C1QBP, DPT, and NRP2, in PASC compared to non-PASC participants, and these differences were significant at an FDR of 0.05 (**Figure 4A**). Additional autoantibodies against immune cell surface receptors (IL7R, CD69), SARS-CoV-2 entry receptors (ACE2), thrombosis proteins (APOH, TFPI), and an apoptosis factor (TNFRSF11B) were increased in PASC participants at an FDR of 0.1, while autoantibodies against the tumor protein, p53, was increased in non-PASC participants (**Figure 4A**). No differences in antibodies against SARS-CoV-2 or other common viruses were observed.

Additionally, epigenetic and transcriptional differences between PASC and non-PASC participants in the full cohort were predominantly in B cells, as was observed in the hospitalized cohort only, with most differences occurring in the DN1 B cell subset (**Figure 4B**). A similar profile in DN1 B cells was observed in PASC participants, with DEGs related to increased activation (e.g., *CD69* and *FCER2*) and anti-viral immunity (e.g., *CD9* and *TRIM5*) being upregulated in PASC participants. A DN1 DAR proximal to TRIM5 was also increased, indicating increased chromatin accessibility to this anti-viral gene in DN1 B cells of PASC participants. A smaller number of gene differences were observed in central and atypical memory B cells, transitional B cells, and CD56 low NK cells (**Figure 4C**).

## Discussion

Infection with SARS-CoV-2 can lead to post-acute sequelae that persists for weeks, months, and even years following infection (3, 44–46). A combination of host and virus factors are thought to be associated with the pathogenesis of PASC, including the persistence of viral antigens, microvascular dysfunction, gut dysbiosis, chronic inflammation, and autoreactive immune responses (6, 7). A limited number of studies have investigated the molecular mechanisms of PASC as it relates to the acute phase of SARS-CoV-2 infection (6, 47, 48). Using multi-omic immune profiling, we sought to investigate early differences in immune responses to SARS-CoV-2 in individuals who eventually did and did not develop PASC.

Our study is unique in that we were able to collect blood samples from COVID-19 patients early in the pathogenesis of disease, with > 90% of samples collected less than a week from the start of COVID-19. Overall, we found a small set of early immune differences in PASC and non-PASC individuals within the first week of COVID-19 disease, with significant molecular signals occurring predominantly in double-negative B cells. The lack of a more robust signal may reflect the heterogeneity in mechanisms underlying PASC and the diverse manifestations of PASC symptoms (49), but our findings suggest that there may be some common immune-mediated mechanisms that begin to influence the ultimate development of PASC even during the acute stage of SARS-CoV-2 infection. We have not examined whether these same signatures continue to differentiate PASC and non-PASC individuals during recovery from acute infection and beyond, but our results suggest that longitudinal monitoring of B cell responses could have value in better understanding and managing PASC.

Previous studies have reported significant molecular differences between individuals hospitalized for COVID-19 compared to those who were not hospitalized, with early immune mechanisms being capable of differentiating trajectories of mild, moderate, and severe COVID-19 (50–53). We similarly observed large differences in the immune response between hospitalized and non-hospitalized COVID-19 patients in their epigenetic and transcriptional signatures, their frequency of cell subsets, and their production of inflammatory cytokines. These findings support previous literature and indicate that within the first week of SARS-CoV-2 pathogenesis, differences in immune responses can differentiate SARS-CoV-2 infected individuals on different disease trajectories.

To reduce the heterogeneity observed among participants, we thus stratified participants based on hospitalization to account for the different follow-up strategies for more severe COVID-19 that may influence the likelihood of developing PASC. As a majority (77%) of our analyzed participants were hospitalized, we did not analyze differences in PASC and non-PASC participants in non-hospitalized patients, given the small sample size for the comparisons in this group (N=13 PASC; N=41 non-PASC). We observed increases in a small number of autoantibodies in hospitalized PASC compared to hospitalized non-PASC participants. Previous studies have found increased autoantibodies to be associated with COVID-19 and PASC (6, 54–60), though the findings associating PASC and autoantibodies have been inconsistent (61). Additionally, prior autoimmunity and antibody cross-reactivity between tissue proteins and SARS-CoV-2 antigens could also be contributing to the pathophysiology of COVID-19, and hence, PASC (62, 63). We did not observe any significant differences in antibodies against SARS-CoV-2 or other common viral pathogens between hospitalized PASC and non-PASC participants, indicating that hospitalized PASC participants likely have a functional acute immune response against the SARS-CoV-2 virus, similar to that of hospitalized non-PASC participants. Similarly, other studies did not find an association between PASC and acute antibody titers against the spike surface protein of SARS-CoV-2 (47, 64), though decreased total acute antibody titers against SARS-CoV-2 were able to predict the development of PASC symptoms (47). We did not have samples collected prior to SARS-CoV-2 infection to assess whether the presence of prior autoantibodies or viral antibodies in our cohort are associated with the development of PASC, which would require further investigation.

We additionally observed an increased interferon signature in hospitalized PASC participants compared to hospitalized non-PASC participants. In particular, numerous ISGs were upregulated in the DN1 B cell subset in PASC participants, and interferon signaling pathways were also increased in a number of effector B and T cell subsets. While interferon signaling plays a critical role in the defense against SARS-CoV-2 during acute infection (65, 66), persistent expression of interferons can lead to inflammatory damage to organ systems and may be linked to autoimmunity (29, 30, 54, 67), thereby contributing to the development of PASC (8, 68). While we have only analyzed the acute immune response to SARS-CoV-2 in PASC and non-PASC individuals, longer term monitoring of interferon responses in individuals with and without PASC could further elucidate whether sustained interferon signaling and pathways may be contributing to the development of PASC in hospitalized patients.

We also observed epigenetic and transcriptional differences in B cells emerging between hospitalized PASC and non-PASC participants early in infection, which may be linked to the increased autoantibodies that we observed in PASC participants. Previous studies have observed dysregulated B cells and new autoreactivity in patients with more severe acute COVID-19 (69–73), which could also be related to the observation that PASC is more common in individuals who experience more severe acute COVID-19 (23, 24). The epigenetic differences we observed in hospitalized PASC versus non-PASC participants were predominantly found in the DN1 subset of DN B cells. In recent years, DN B cells have been suggested to play important roles in cancers, infections, and autoimmune diseases (74), though their function in these contexts remain unclear. Double-negative B cells make up approximately 5% of PBMCs (75). They are matured, peripheral B cells that lack expression of CD27 and IgD and are thought to be precursors of memory B cells (25), with DN1 cells showing strong transcriptional similarity to class-switched memory B cells (76). Severe COVID-19 has been associated with a decreased frequency of DN1 cells and increased frequencies of DN2 and DN3 cells (70, 72, 77). These changes, however, may be transient, as studies have found that DN2 cells disappear soon after recovery from COVID-19 (70, 78, 79). Changes in DN B cells in the context of PASC, however, remain relatively understudied (15), and the exact function of DN B cells remains unclear. We observed no significant differences in DN1 cell or B cell frequencies between hospitalized PASC and non-PASC participants during their presentation to a hospital for COVID-19. However, it is intriguing that most of the epigenetic and transcriptional differences that we observe were found within DN1 B cells. Additionally, the increase in activation and anti-viral genes in the DN1 subset of hospitalized PASC participants indicate potential priming of this subset compared to non-PASC participants. Long term evaluation of this subset from acute infection to recovery could help elucidate the possible roles of DN1 B cells in the development of PASC.

We also examined molecular differences in PASC and non-PASC participants that were and were not hospitalized for COVID-19 to better understand the immune signals emerging from a broader population. We observed overlapping molecular differences in autoantibodies and DN1 B cells as with the hospitalized PASC and non-PASC cohort only, although a smaller number of significant differences emerged, possibly due to the increased heterogeneity within this group. The mechanisms of PASC may thereby vary according to the severity of acute SARS-CoV-2 infection, which may need to be considered when elucidating the role of the immune system in the development of PASC.

In summary, our analyses provide a detailed examination of the early immune response to COVID-19 and its ability to differentiate individuals on different severity and PASC trajectories. While individuals hospitalized for COVID-19 have significantly different immune responses compared to non-hospitalized individuals early on in SARS-CoV-2 infection, less differences are observed in individuals who do and do not develop PASC. The emerging differences in autoantibody responses and B cell phenotypes in PASC participants, however, are intriguing, in addition to the interferon signatures observed in PASC participants that were hospitalized. Future studies elucidating the function of DN B cells are needed to better understand the contribution and role of these cells in COVID-19, PASC, and other diseases. Together, our data ultimately provides a framework for guiding future research when monitoring longitudinal immune responses in the development of PASC.

## Data availability statement

The datasets presented in this article are not readily available because of limitations protecting proprietary information. Requests to access the datasets should be directed to immuneprofiler@verily.com.

## Ethics statement

The PRESCO study was approved by a central Western Institutional Review Board (Protocol number: 20201016) and at each of the eight sites. The studies were conducted in accordance with the local legislation and institutional requirements. The participants provided their written informed consent to participate in this study.

## Author contributions

JL: Data curation, Formal Analysis, Writing – original draft, Writing – review & editing. MW: Data curation, Formal Analysis, Writing – original draft, Writing – review & editing. PK: Data curation, Formal Analysis, Writing – review & editing. CC: Conceptualization, Writing – review & editing. KD: Conceptualization, Writing – review & editing. GT: Data curation, Formal Analysis, Writing – review & editing. VR: Conceptualization, Writing – review & editing. HZ: Conceptualization, Writing – review & editing. WC: Conceptualization, Writing – review & editing. NH: Data curation, Formal Analysis, Writing – review & editing. JA: Data curation, Formal Analysis, Writing – review & editing. CW: Data curation, Formal Analysis, Writing – review & editing. CP: Data curation, Formal Analysis, Writing – review & editing. IM: Data curation, Formal Analysis, Writing – review & editing. VT: Investigation, Writing – review & editing. JM: Investigation, Writing – review & editing. CD: Investigation, Writing – review & editing. IR: Investigation, Writing – review & editing. MB: Investigation, Writing – review & editing. JK: Investigation, Writing – review & editing. SP: Formal analysis, Investigation, Writing – review & editing. BP: Formal analysis, Writing – review & editing. MS: Formal analysis, Investigation, Writing – review & editing. CK: Conceptualization, Writing – original draft, Writing – review & editing.
