# Supplementary material for: Early immune factors associated with the development of post-acute sequelae of SARS-CoV-2 infection in hospitalized and non-hospitalized individuals
Source: Front Immunol. 2024 Jan 22;15:1348041. doi: 10.3389/fimmu.2024.1348041 (PMC10838987; doi:10.3389/fimmu.2024.1348041)
Supplement: Supplementary file 1 [file DataSheet_1.docx]

***Supplementary Material***

**Early immune factors associated with the development of post-acute sequelae of SARS-CoV-2 infection in hospitalized and non-hospitalized individuals**

Jacqueline M. Leung*^1^, Michelle J. Wu*^1^, Pouya Kheradpour*^1^, Chen Chen^1^, Katherine A. Drake^1^, Gary Tong^1^, Vanessa K. Ridaura^1^, Howard C. Zisser^1^, William A. Conrad^2^, Natalia Hudson^3^, Jared Allen^3^, Christopher Welberry^3^, Celine Parsy-Kowalska^3^, Isabel Macdonald^3^, Victor F. Tapson^4^, James N. Moy^5^, Christopher R. deFilippi^6^, Ivan O. Rosas^7^, Mujeeb Basit^8^, Jerry A. Krishnan^9^, Sairam Parthasarathy^10^, Bellur S. Prabhakar^11^, Mirella Salvatore^12^, Charles C. Kim^1^

* These authors share first authorship

**Correspondence:**

Charles C. Kim

charliekim@verily.com

**ACKNOWLEDGEMENTS**

We thank all the participants and study staff that were involved in this study during a challenging time when COVID-19 was first emerging.

| **Acknowledged individual** | **Role** | **Affiliation** |
| --- | --- | --- |
| Benazir Khan, MD | Recruitment and retention of research participants | Baylor College of Medicine |
| Fernando Poli, MD | Recruitment and retention of research participants | Baylor College of Medicine |
| Rafael Cardenas Castillo, MD | Recruitment and retention of research participants | Baylor College of Medicine |
| Antonina Caudill, MPH | Recruitment and retention of research participants, collected data | Cedars-Sinai Medical Center |
| Cristabelle Ojukwu, BS | Sample processing | Cedars-Sinai Medical Center |
| Devin Fine, BS | IRB and regulatory issues | Cedars-Sinai Medical Center |
| Emad Bayoumi, MD, MBA | Recruitment and retention of research participants, collected data | Cedars-Sinai Medical Center |
| Ethan Pascual, MA | Recruitment and retention of research participants, collected data | Cedars-Sinai Medical Center |
| Gregg Clapham, MA | Administrative tasks, Inventory | Cedars-Sinai Medical Center |
| Lisa Herrera | Administrative tasks | Cedars-Sinai Medical Center |
| Millie Gomez, MD | Budget Coordinator | Cedars-Sinai Medical Center |
| Po-En Chen, RN, BSN | Recruitment and retention of research participants, collected data | Cedars-Sinai Medical Center |
| Sara Langley, RN, MSN | Recruitment and retention of research participants, collected data | Cedars-Sinai Medical Center |
| Susan Jackman, RN, MS | Recruitment and retention of research participants, collected data, IRB and regulatory issues, budget and contract coordinator | Cedars-Sinai Medical Center |
| Tabia Richardson, MPH, PhD | Supervised team | Cedars-Sinai Medical Center |
| Treasure Joyce | Logistics organization | Providence Little Company of Mary Medical Center Torrance |
| Amy Gosha, BSN | Recruitment and retention of research participants, collected data | Rush University Medical Center |
| Heidi Erickson, RN | Recruitment and retention of research participants, collected data, IRB and regulatory issues, critically reviewed the study proposal | University of Arizona |
| Anjana Maheswaran | Coordinator | University of Illinois Chicago |
| Dawood Darbar, MBChB, MD | Investigator | University of Illinois Chicago |
| Heather Prendergast, MD | Investigator | University of Illinois Chicago |
| Jan Kitajewski, PhD | Investigator | University of Illinois Chicago |
| Janet Lin, MD | Site PI | University of Illinois Chicago |
| Jeff Jacobson, MD | Investigator | University of Illinois Chicago |
| Jonathan Klein, MD | Investigator | University of Illinois Chicago |
| Julie DeLisa, MS | Director, Regulatory Affairs | University of Illinois Chicago |
| Lauren Castro, RN | Research Nurse and Nurse Manager | University of Illinois Chicago |
| Lourdes Norwick, RN | Research Nurse | University of Illinois Chicago |
| Marina Del Rios, MD | Investigator | University of Illinois Chicago |
| Patricia Finn, MD | Investigator | University of Illinois Chicago |
| Pavitra Kotini-Shah MD | Investigator | University of Illinois Chicago |
| Research staff in the Emergency Department, inpatient units, and laboratories at University of Illinois Chicago | Conducted study | University of Illinois Chicago |
| Richard Novak, MD | Investigator | University of Illinois Chicago |
| Sai Illendula, MS | Data Manager | University of Illinois Chicago |
| Shaveta Khosla | Coordinator | University of Illinois Chicago |
| Terry Vanden Hoek, MD | Investigator | University of Illinois Chicago |
| Wendy Haase, MBA | Project Manager | University of Illinois Chicago |
| Yining Chen | Coordinator | University of Illinois Chicago |
| Richard Medford, MD | Site PI | University of Texas Southwestern Medical Center |
| Samuel McDonald MD, MS | Site PI | University of Texas Southwestern Medical Center |
| Biostatistics Team | Managed and analyzed clinical data | Verily Life Sciences |
| Clinical Data Management team | Co-ordinated sites, managed clinical data | Verily Life Sciences |
| Clinical Operations Team | Co-ordinated sites, managed clinical data | Verily Life Sciences |
| Leera Choi | Co-ordinated sites, contract coordinator, IRB, study initiation | Verily Life Sciences |
| Molecular Production team | Sample processing | Verily Life Sciences |
| Weill Cornell Medicine Clinical & Translational Science Center | Provided study support | Weill Cornell Medicine Clinical & Translational Science Center |
| Anna Gwak, BA | IRB and regulatory issues | Weill Cornell Medicine Transplantation-Oncology Infectious Diseases Clinical Research Unit |
| Elizabeth Salsgiver, BS, MPH | Budget and contract coordinator, logistics organization | Weill Cornell Medicine Transplantation-Oncology Infectious Diseases Clinical Research Unit |
| Jack Spagnoletti, BA | Recruitment and retention of research participants, collected data, sample processing | Weill Cornell Medicine Transplantation-Oncology Infectious Diseases Clinical Research Unit |

**SUPPLEMENTARY METHODS**

**Study oversight**

The Predictors of Severe COVID-19 Outcomes (PRESCO) study was conducted at eight institutions across the United States and was approved by a central WCG Institutional Review Board (WCG IRB Protocol # 20201016) and each academic institution (Trial Registration Number: NCT04388813). The WCG IRB Protocol number was utilized by all sites unless stated otherwise (The University of Arizona, Cedars-Sinai Medical Center, University of Illinois at Chicago, Rush University Medical Center (IRB #20050404-IRB02), Weill Cornell Medical College (IRB #20-08022553), University of Texas Southwestern Medical Center, Baylor College of Medicine, and Inova Health Care Services). All participants or their legally authorized representatives provided written informed consent.

**Participant recruitment**

Participants were recruited into the PRESCO study from hospitalized and non-hospitalized (ambulatory) settings. For hospitalized patients, a partial HIPAA waiver from the Institutional Review Board was obtained at all of the sites. Clinical research coordinators in the PRESCO study assessed new admission patient lists and from these patients, selected those that were COVID-19 positive cases. After receiving permission from the attending physician, patients, and/or their surrogates (in the event that the potential participant was unable to give informed consent), participants were approached by the clinical research coordinators, who explained the purpose and risks for participation in the PRESCO study as part of the informed consenting process. For ambulatory patients, flyers regarding the PRESCO study were posted in urgent care facilities, COVID-19 testing centers, and ambulatory clinics. These flyers indicated the nature and purpose of the PRESCO study. Potential patients who tested positive for SARS-CoV-2 infection called the clinical research coordinators’ phone numbers that were listed on the flyers. Additionally, providers who were caring for patients in clinics and other facilities could also refer potential participants to the study if their patients exhibited symptoms of COVID-19 and tested positive.

**Quantification of autoantibodies and antibodies against viral and bacterial antigens**

## Proteins and peptides

Individual antigenic protein DNA was either amplified from IMAGE clones (Source BioScience, Nottingham, UK) or designed in silico and synthesized chemically (Life Technologies, Carlsbad, USA or ThermoFisher, Waltham, USA) and cloned into the expression vector pQE30-NST or the pET21b vector modified to encode a biotin tag and a hexa-histidine-tag to aid in purification of the expressed protein.

Recombinant gene expression was performed under control of the lactose inducible promoter in either *E. coli* SCS1 cells carrying plasmid pSE111 or BL21(DE3)RIPL (Agilent). Cells were cultivated in auto-induction medium (Overnight Express auto-induction medium, Merck, Darmstadt) overnight and harvested by centrifugation. Bacterial pellets were lysed by resuspension in lysis buffer (BugBuster Master Mix, Merck, Burlington, USA) and/or 6 M guanidinium-HCl.

Soluble proteins were affinity-purified after binding to nickel resin columns (Macherey-Nagel, Düren, Germany, Cytiva, Marlborough, USA), following manufacturer’s protocols.

A full list of the proteins used in this study can be found in Supplementary Table 2 for human proteins and Supplementary Table 3 for viral proteins. A subset (n=81) of human and viral antigens were procured externally from various sources. Lyophilized proteins were resuspended according to manufacturer’s recommendations.

Peptides of putative viral epitopes were obtained through custom synthesis by ProteoGenix, Schiltingheim, France. Lyophilized 1mg aliquots were resuspended in 100 µl of ACN:H2O=1:3, ACN:H2O=1:2 or 6.5 M Urea. The list of peptides used can be found in Supplementary Table 5.

## Immobilisation of proteins and peptides

Proteins were immobilized onto magnetic MagPlex™ microspheres (Luminex Corporation, Austin, TX, USA) as previously described [(1)](https://paperpile.com/c/w9cRFj/n3YN). Multiplex arrays were produced by pooling up to 227 regions. Peptides were immobilized onto magnetic MagPlex™ microspheres (Luminex Corporation, Austin, TX, USA) in a reaction based on the standard manufacturer's protocol for peptide coupling [(2)](https://paperpile.com/c/w9cRFj/xuTa). Control antigens for peptide panels were coupled as described and a multiplex bead-based array was produced by pooling up to 198 peptides with the control protein-coupled beads.

## Multiplexed Assays

Multiplexed bead-based arrays were assembled with a total of 744 antigens: 441 human proteins indicated in immune responses (and including 3 Ig controls), 114 viral proteins that included differing recombinant versions of proteins of SARS-CoV-2 as well as other viruses (MERS-CoV, SARS-CoV-1, HCoV-HKU1, HCoV-NL63, HCoV-OC43, HCoV-229E, SARS-CoV-2, Influenza A, Influenza B), and 192 viral peptides of which 178 originated from SARS-CoV-2 sequences and 14 were from other viruses.

A summary of the proteins selected for screening of COVID-19 patient samples is presented in Supplementary Table 4, and a summary of the peptides selected is presented in Supplementary Table 6. Assay methodology has been described previously [(3)](https://paperpile.com/c/w9cRFj/y3SP). Briefly, for analysis of human autoantigens and viral proteins a 1:100 or 1:800 serum dilution respectively was prepared in 96-well plates. The serum dilutions were first incubated for 20 minutes to neutralize any human IgG eventually directed against *E. coli* proteins. For analysis of viral peptides, a 1:100 serum dilution was 96-well plates. Homogenized bead arrays were combined with pre-diluted serum dilutions and incubated for 22 hours (900 rpm, +2 to +8 °C). Beads were washed and a secondary R-phycoerythrin-labeled antibody (5 μg/ml, goat anti-human, Dianova, Hamburg, Germany) was added for 60 minutes (900 rpm, RT). Subsequently, after washing, beads were resuspended in sheath fluid (Luminex Corporation, Austin, Texas) and analyzed in a FlexMap3D (Luminex Corporation, Austin, Texas) device for fluorescent signal readout. The binding events were displayed as median fluorescence intensity (MFI). Measurements were disregarded when low numbers of bead events (<10 beads) were counted per bead region. Median intra- and inter-plate Coefficients of Variation (CV) were calculated by measuring three reference samples: one COVID-19 positive, one Systemic Lupus erythematosus (SLE) positive and one SLE and COVID-19 negative.

## Quality control of assay data

## Several fixed control criteria were defined to guarantee high data quality. Data completeness threshold was set at >98% of available instrument data. Additionally bead count statistics were controlled to not allow more than 10% drop-outs. This criterion prevented use of MFI values for plates, samples, and antigens with insufficient bead counts. The lower MFI range was monitored via median MFI of BSA-coupled beads and was set to be below 500. Upper median MFI range of the IgG-coupled beads was set to >20,000. The antigen panel was divided into 4 bead-based arrays for ease of processing of up to 230 bead regions. Assays contained control reference samples as well as sample-antigen pairs measured in triplicate in each plate. This allowed for control of intra- and inter- plate variance which were both set to <30%. Additionally for proteins raised in *E. coli*, background reactivity of sera to *E. coli* proteins was monitored.

**REFERENCES**

1. [Budde P, Zucht H-D, Vordenbäumen S, Goehler H, Fischer-Betz R, Gamer M, Marquart K, Rengers P, Richter J, Lueking A, et al. Multiparametric detection of autoantibodies in systemic lupus erythematosus. *Lupus* (2016) 25:812–822.](http://paperpile.com/b/w9cRFj/n3YN)

2. [Angeloni S, Cordes R, Dunbar S, Garcia C, Gibson G, Martin C, Stone V. xMAP cookbook: a collection of methods and protocols for developing multiplex assays with xMAP technology. *Luminex: Austin, TX, USA*](http://paperpile.com/b/w9cRFj/xuTa)

3. [Liu Y, Ebinger JE, Mostafa R, Budde P, Gajewski J, Walker B, Joung S, Wu M, Bräutigam M, Hesping F, et al. Paradoxical sex-specific patterns of autoantibody response to SARS-CoV-2 infection. *J Transl Med* (2021) 19:524.](http://paperpile.com/b/w9cRFj/y3SP)

**SUPPLEMENTARY FIGURES**

**Supplementary Figure 1. Overlap of participants used for various assays in the PRESCO study for the hospitalized and non-hospitalized PASC and non-PASC comparisons.** Venn diagram showing the overlap of participants used for multi-omic profiling (flow cytometry, ATAC-seq, RNA-seq, and TaPE-seq), plasma cytokine detection by Luminex, and plasma autoantibody/antibody detection in the hospitalized and non-hospitalized PASC and non-PASC comparisons.


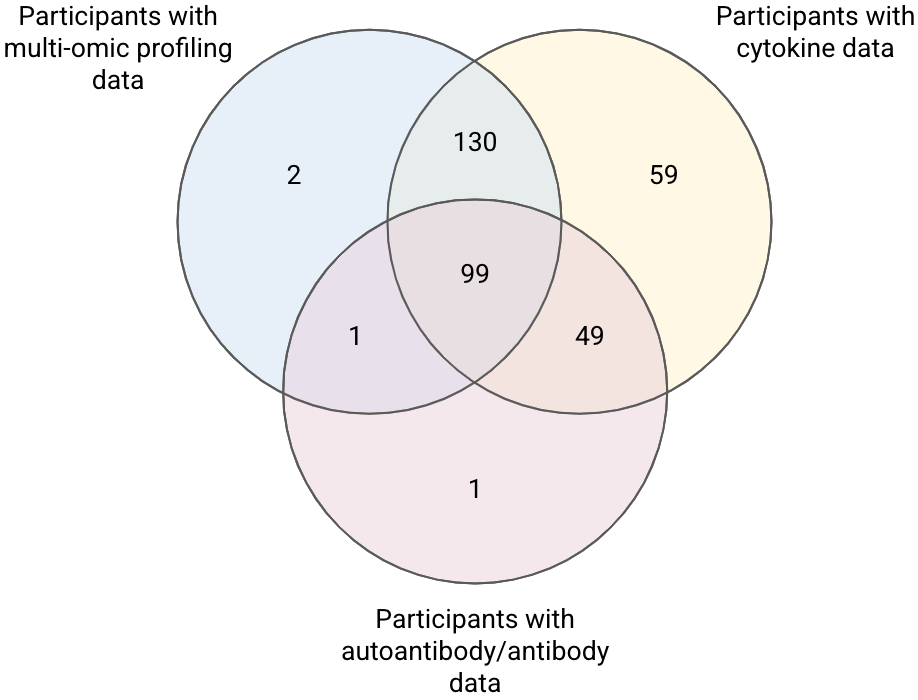


**Supplementary Figure 2. Workflow describing the process for multi-omic profiling.** Abbreviations for immune cell subsets are defined in Supplemental Table 1.


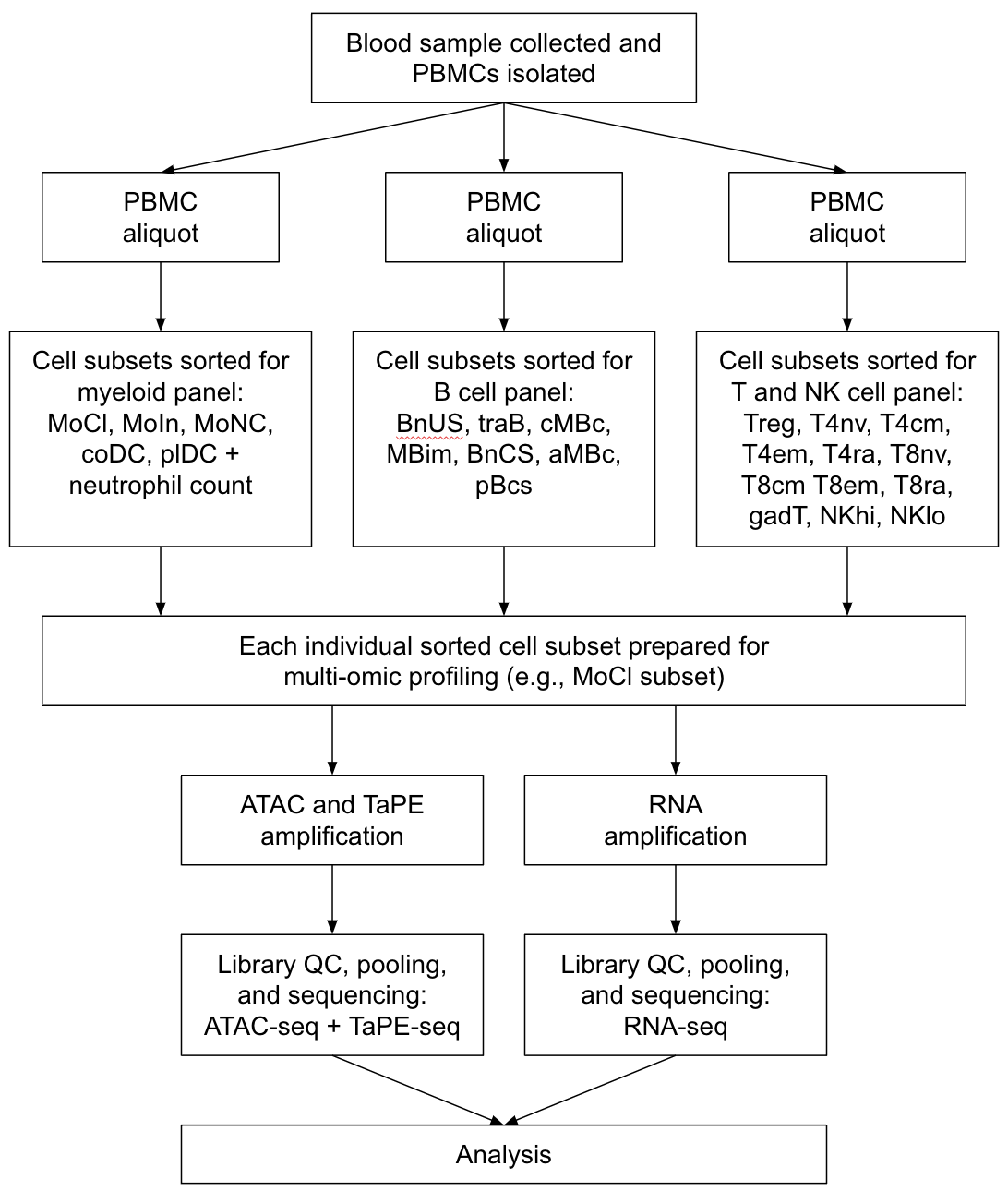


**Supplementary Figure 3. Immune cell frequencies in the blood of hospitalized PASC and hospitalized** **non-PASC participants.** Box plots of cell subset frequencies grouped by hospitalized PASC and hospitalized non-PASC participants during their presentation to the hospital. Abbreviations for immune cell subsets are defined in Supplemental Table 1.


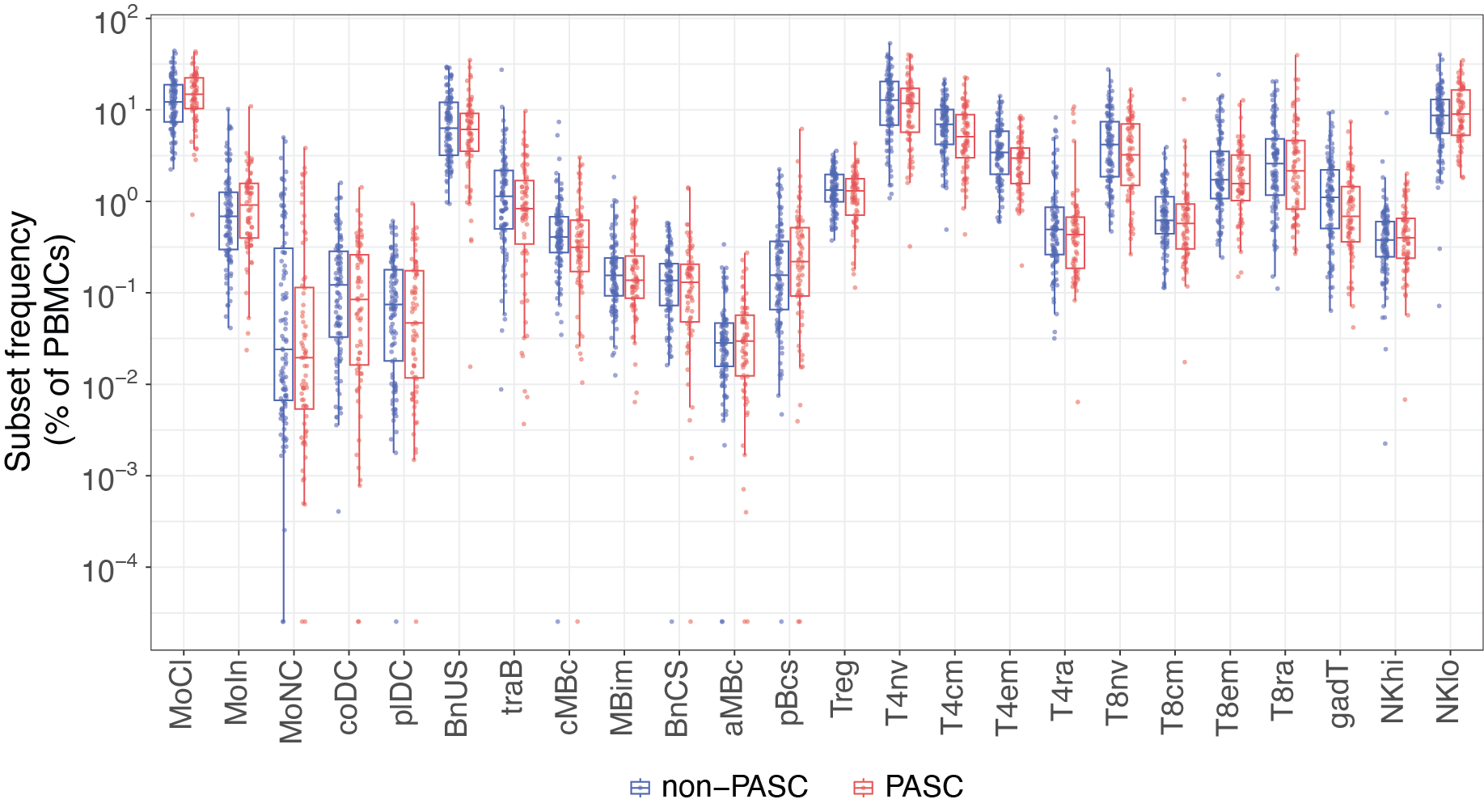


**Supplementary Figure 4. Cytokine and chemokine concentrations in the blood of hospitalized PASC and hospitalized non-PASC participants.** Box plots of cytokine concentrations in pg/ml grouped by hospitalized PASC and hospitalized non-PASC participants during their presentation to the hospital.


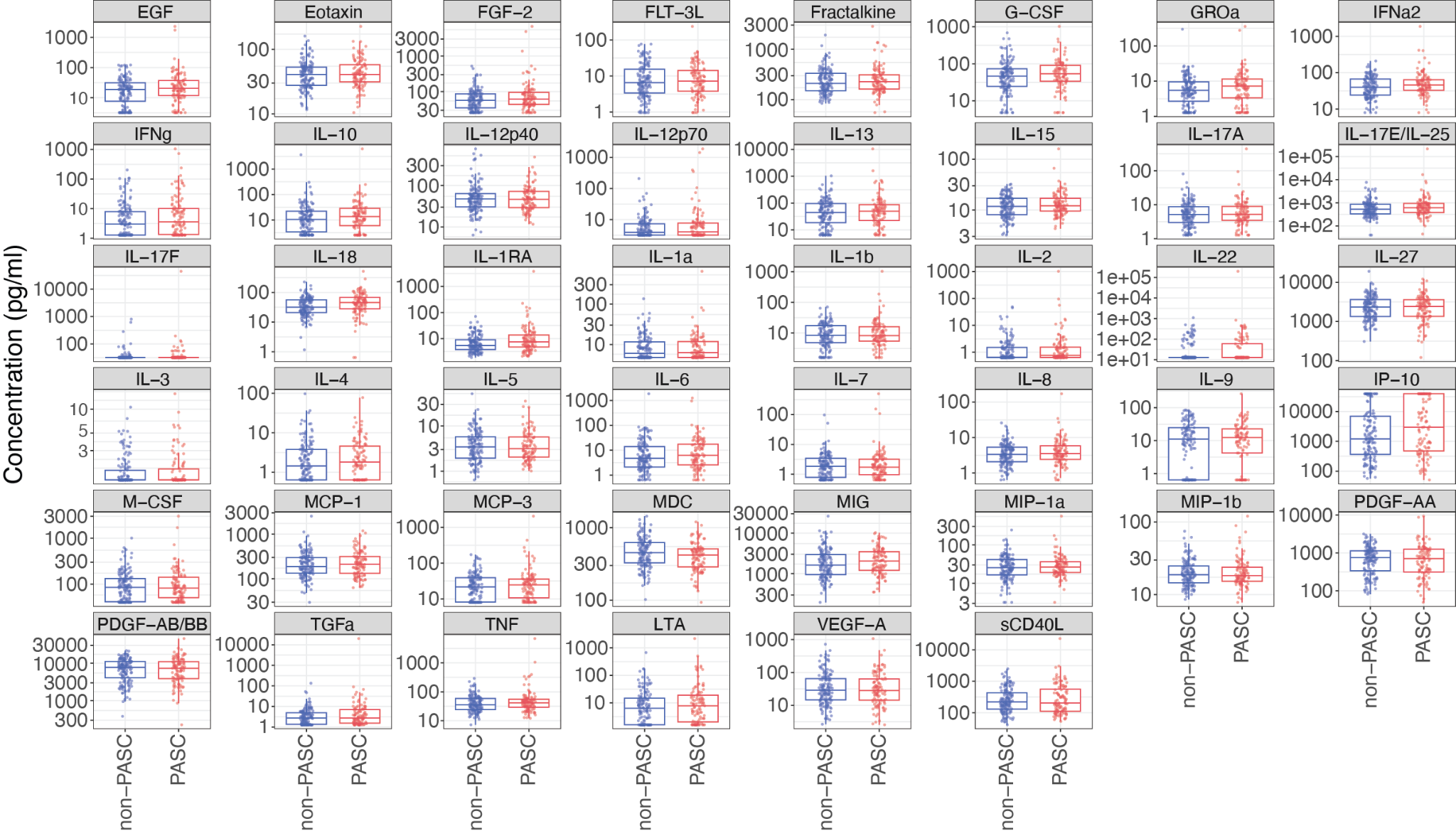


**SUPPLEMENTARY TABLES**

**Supplementary Table 1. Immune cell types profiled.** The 24 immune cell subsets profiled for multi-omic analysis.


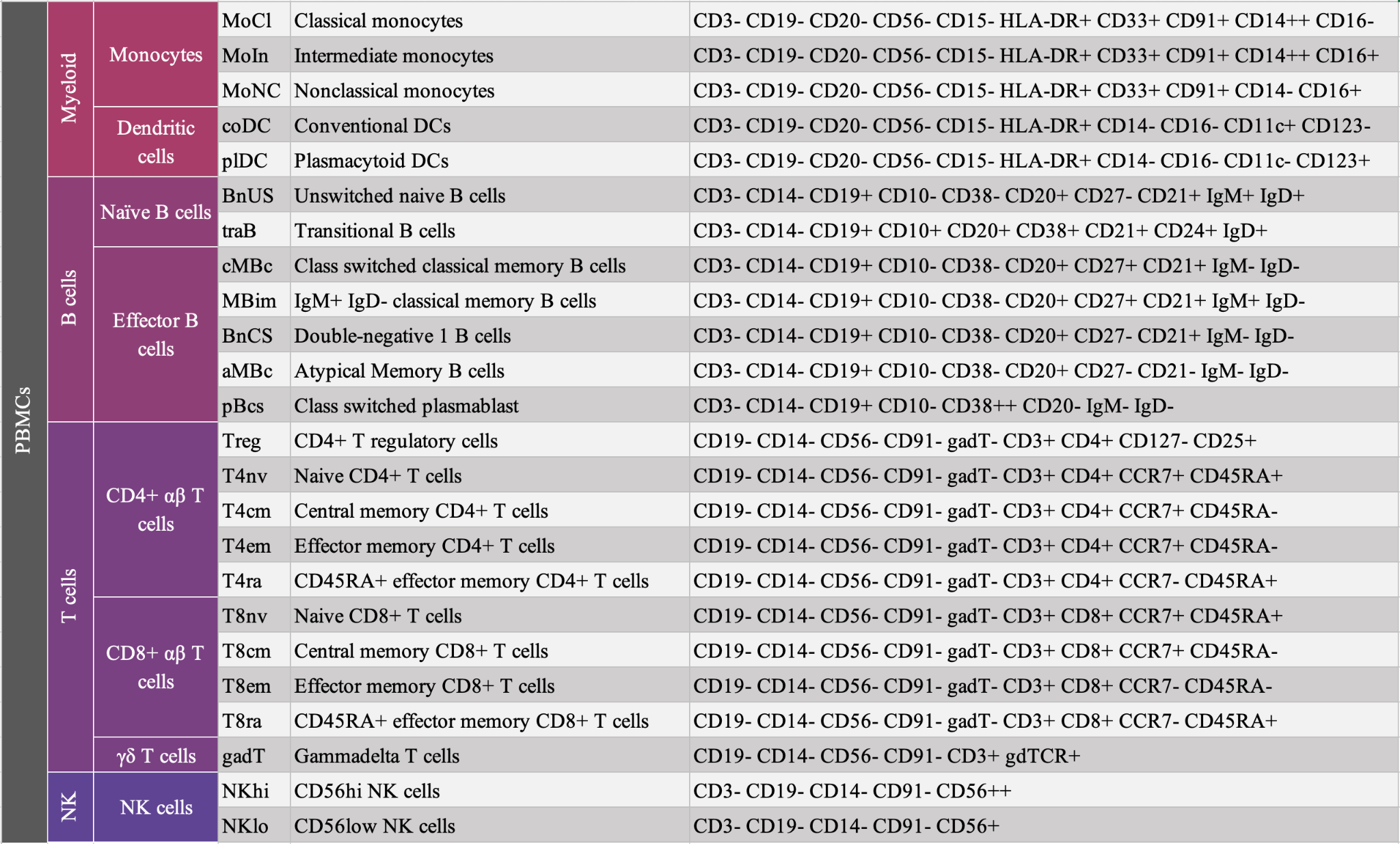


**Supplementary Table 2: List of antigens used in human protein bead arrays.**

| **Symbol** | **Target Name** | **Gene ID** |
| --- | --- | --- |
| AARS | Alanyl-tRNA synthetase | 16 |
| ABO | Alpha 1-3-N-acetylgalactosaminyltransferase and alpha 1-3-galactosyltransferase | 28 |
| ACE2 | Angiotensin-converting enzyme 2 | 59272 |
| ADAMTS13 | ADAM metallopeptidase with thrombospondin type 1 motif 13 | 11093 |
| ADRA1A | Adrenoceptor alpha 1A | 148 |
| ADGRE5 | Adhesion G Protein-Coupled Receptor E5 | 976 |
| ADRB2 | Adrenoceptor beta 2 | 154 |
| AK4 | Adenylate Kinase 4 | 205 |
| ANPEP | Alanyl aminopeptidase, membrane (Aminopeptidase N) | 290 |
| ANXA2 | Annexin II | 302 |
| ANXA5 | Annexin V | 308 |
| APOA4 | Apolipoprotein A4 | 337 |
| APOH | Apolipoprotein H | 350 |
| AQP4 | Aquaporin 4 | 361 |
| BCAP31 | B Cell Receptor Associated Protein 31 | 10134 |
| BICD2 | BICD cargo adaptor 2 | 23299 |
| BLMH | Bleomycin hydrolase | 642 |
| BMP1 | Bone morphogenetic protein 1 | 649 |
| BMP4 | Bone morphogenetic protein 4 | 652 |
| BMPR2 | Bone morphogenetic protein receptor type 2 | 659 |
| BPI | Bactericidal/permeability-increasing protein | 671 |
| BSG | Basigin | 682 |
| BTBD7 | BTB domain containing 7 | 55727 |
| C1QA | Complement C1q A chain | 712 |
| C1QB | Complement C1q B chain | 713 |
| C1QBP | Complement C1q binding protein | 708 |
| C3 | Complement C3 | 718 |
| C4A | Complement C4A (Rodgers blood group) | 720 |
| CADM3 | Cell Adhesion Molecule 3 | 57863 |
| CAGE | DEAD-Box Helicase 53 | 168400 |
| CALCA | Calcitonin Related Polypeptide Alpha | 796 |
| CCL11 | C-C motif chemokine ligand 11 | 6356 |
| CCL14 | C-C motif chemokine ligand 14 | 6358 |
| CCL15 | C-C Motif Chemokine Ligand 15 | 6359 |
| CCL2 | C-C motif chemokine ligand 2 | 6347 |
| CCL21 | C-C motif chemokine ligand 21 | 6366 |
| CCL25 | C-C motif chemokine ligand 25 | 6370 |
| CCL27 | C-C motif chemokine ligand 27 | 10850 |
| CCL4 | C-C motif chemokine ligand 4 | 6351 |
| CCL5 | C-C motif chemokine ligand 5 | 6352 |
| CCL7 | C-C motif chemokine ligand 7 | 6354 |
| CCL8 | C-C motif chemokine ligand 8 | 6355 |
| CCR7 | C-C motif chemokine receptor 7 | 1236 |
| CD101 | CD101 molecule | 9398 |
| CD14 | CD14 molecule | 929 |
| CD160 | CD160 molecule | 11126 |
| CD163 | CD163 molecule | 9332 |
| CD24 | CD24 molecule | 100133941 |
| CD244 | CD244 molecule | 51744 |
| CD27 | CD27 molecule | 939 |
| CD274 | CD274 molecule | 29126 |
| CD276 | CD276 molecule | 80381 |
| CD28 | CD28 molecule | 940 |
| CD38 | CD38 molecule | 952 |
| CD4 | CD4 molecule | 920 |
| CD40 | CD40 molecule | 958 |
| CD40LG | CD40 ligand | 959 |
| CD44 | CD44 molecule | 960 |
| CD47 | CD47 molecule | 961 |
| CD48 | CD48 molecule | 962 |
| CD5L | CD5 molecule like | 922 |
| CD68 | CD68 molecule | 968 |
| CD69 | CD69 molecule | 969 |
| CD74 | CD74 molecule | 972 |
| CD80 | CD80 molecule | 941 |
| CD86 | CD86 molecule | 942 |
| CD8A | CD8a molecule | 925 |
| CD99 | CD99 molecule | 4267 |
| CD99L2 | CD99 molecule like 2 | 83692 |
| CDK1 | Cyclin dependent kinase 1 | 983 |
| CEACAM1 | Carcinoembryonic antigen related cell adhesion molecule 1 | 634 |
| CENPB | Centromere protein B | 1059 |
| CENPC | Centromere protein C | 1060 |
| CENPH | Centromere Protein H | 64946 |
| CENPJ | Centromere Protein J | 55835 |
| CHD3 | Chromodomain helicase DNA binding protein 3 | 1107 |
| Chd4 | Chromodomain helicase DNA binding protein 4 | 1108 |
| CHD8 | Chromodomain helicase DNA binding protein 8 | 57680 |
| CHGA | Chromogranin A | 1113 |
| CHGB | Chromogranin B | 1114 |
| CHRM3 | Cholinergic Receptor Muscarinic 3 | 1131 |
| CK20 | Keratin 20 | 54474 |
| COL1A1 | Collagen alpha-1 (I) chain | 1277 |
| CRP | C-reactive protein | 1401 |
| CRYAB | Crystallin Alpha B | 1410 |
| CSF2 | Colony stimulating factor 2 | 1437 |
| CSF2RA | Colony stimulating factor 2 receptor alpha subunit | 1438 |
| CSF3 | Colony stimulating factor 3 | 1440 |
| CSTB | Cystatin B | 1476 |
| CTAG1B | Cancer/Testis Antigen 1B | 1485 |
| CTLA4 | Cytotoxic T-lymphocyte associated protein 4 | 1493 |
| CTSB | Cathepsin B | 1508 |
| CTSG | Cathepsin G | 1511 |
| CTSL | Cathepsin L | 1514 |
| CX3CL1 | C-X3-C motif chemokine ligand 1 | 6376 |
| CXCL1 | C-X-C motif chemokine ligand 1 | 2919 |
| CXCL10 | C-X-C Motif Chemokine Ligand 10 | 3627 |
| CXCL12 | C-X-C motif chemokine ligand 12 | 6387 |
| CXCL13 | C-X-C Motif Chemokine Ligand 13 | 10563 |
| CXCL16 | C-X-C motif chemokine ligand 16 | 58191 |
| CXCL2 | C-X-C motif chemokine ligand 2 | 2920 |
| CXCL3 | C-X-C motif chemokine ligand 3 | 2921 |
| CXCL8 | C-X-C Motif Chemokine Ligand 8 | 3576 |
| CXCL9 | C-X-C Motif Chemokine Ligand 9 | 4283 |
| DBT | Dihydrolipoamide branched chain transacylase E2 | 1629 |
| DDX58 | RNA Sensor RIG-I | 23586 |
| DHODH | Dihydroorotate dehydrogenase | 1723 |
| DLAT | Dihydrolipoamide S-acetyltransferase | 1737 |
| DLST | Dihydrolipoamide S-succinyltransferase | 1743 |
| DPP4 | Dipeptidyl peptidase 4 | 1803 |
| DPT | Dermatopontin | 1805 |
| ECE1 | Endothelin converting enzyme 1 | 1889 |
| EHD1 | EH domain containing 1 | 10938 |
| EIF2AK2 | Eukaryotic translation initiation factor 2 alpha kinase 2 | 5610 |
| EIF4H | Eukaryotic Translation Initiation Factor 4H | 7458 |
| EIF5 | Eukaryotic translation initiation factor 5 | 1983 |
| ELANE | Elastase, neutrophil expressed | 1991 |
| ENO1 | Enolase 1 | 2023 |
| ENO2 | Enolase 2 | 2026 |
| EXOSC10 | Exosome component 10 | 5394 |
| F9 | Coagulation factor IX | 2158 |
| F10 | Coagulation factor X | 2159 |
| F11 | Coagulation factor XI | 2160 |
| F2 | Coagulation factor II, thrombin | 2147 |
| F2R | Thrombin Receptor Protein | 2149 |
| F8 | Coagulation factor VIII | 2157 |
| FAF1 | Fas associated factor 1 | 11124 |
| Fas | Fas cell surface death receptor | 355 |
| FasLG | Fas ligand (TNF superfamily, member 6) | 356 |
| FCGR1A | Fc fragment of IgG, high affinity Ia, receptor | 2209 |
| FCGR2A | Fragment C gamma receptor 2A | 2212 |
| FCGR2B | Fc Gamma Receptor IIb | 2213 |
| FCGR3A | Fc Gamma Receptor IIIa | 2214 |
| FGA | Fibrinogen alpha chain | 2243 |
| FGB | Fibrinogen beta chain | 2244 |
| FGF1 | Fibroblast growth factor 1 | 2246 |
| FGF19 | Fibroblast growth factor 19 | 9965 |
| FGF-2 | FGF-2 | 2247 |
| FGF21 | Fibroblast growth factor 21 | 26291 |
| FGFBP2 | Fibroblast growth factor binding protein 2 | 83888 |
| FGFR1 | Fibroblast growth factor receptor 1 | 2260 |
| FGG | Fibrinogen gamma chain | 2266 |
| FSCN1 | Fascin actin-bundling protein 1 | 6624 |
| FURIN | Furin | 5045 |
| GAD65 | Glutamate Decarboxylase 2 | 2572 |
| GARS | Glycyl-tRNA synthetase | 2617 |
| GDF15 | Growth differentiation factor 15 | 9518 |
| GNLY | Granulysin | 10578 |
| GP2 | Glycoprotein 2 | 2813 |
| GRP | Gastrin releasing peptide | 2922 |
| GZMA | Granzyme A | 3001 |
| GZMB | Granzyme B | 3002 |
| GZMK | Granzyme K | 3003 |
| HARS | Histidyl-tRNA synthetase | 3035 |
| HAVCR2 | Hepatitis A virus cellular receptor 2 | 84868 |
| HGF | Hepatocyte Growth Factor | 3082 |
| HIP1 | Huntingtin interacting protein 1 | 3092 |
| HIST2HB | H2B Clustered Histone 5 | 3017 |
| HIST1H4A | Histone cluster 1 H4 family member a | 8359 |
| HIST2H2AA3 | Histone cluster 2 H2A family member a3 | 8337 |
| HN1L | Haematological and neurological expressed 1 like | 90861 |
| HNRNPA1 | Heterogeneous nuclear ribonucleoprotein A1 | 3178 |
| HNRNPA2B1 | Heterogeneous nuclear ribonucleoprotein A2/B1 | 3181 |
| HNRNPM | Heterogeneous nuclear ribonucleoprotein M | 4670 |
| HSPE1 | Heat shock protein family E (Hsp10) member 1 | 3336 |
| HuD/ELAV4 | ELAV Like RNA Binding Protein 4 | 1996 |
| ICA1 | Islet cell autoantigen 1 | 3382 |
| ICOSLG | Inducible T-cell costimulator ligand | 23308 |
| IFIH1 | Interferon induced with helicase C domain 1 | 64135 |
| IFNA10 | Interferon alpha 10 | 3446 |
| IFNA13 | Interferon alpha 13 | 3447 |
| IFNA14 | Interferon alpha 14 | 3448 |
| IFNA16 | Interferon alpha 16 | 3449 |
| IFNA17 | Interferon Alpha 17 | 3451 |
| IFNA2 | Interferon alpha 2 | 3440 |
| IFNA21 | Interferon alpha 21 | 3452 |
| IFNA4 | Interferon alpha 4 | 3441 |
| IFNA5 | Interferon alpha 5 | 3442 |
| IFNA6 | Interferon alpha 6 | 3443 |
| IFNA8 | Interferon alpha 8 | 3445 |
| IFNAR1 | Interferon alpha and beta receptor subunit 1 | 3454 |
| IFNAR2 | Interferon alpha and beta receptor subunit 2 | 3455 |
| IFNB1 | Interferon beta 1 | 3456 |
| IFNE | Interferon epsilon | 338376 |
| IFNG | Interferon gamma | 3458 |
| IFNGR2 | Interferon gamma receptor 2 (Interferon gamma transducer 1) | 3460 |
| IFNK | Interferon kappa | 56832 |
| IFNL2 | Interferon lambda 2 | 282616 |
| IFNL3 | Interferon lambda 3 | 282617 |
| IFNW1 | Interferon omega 1 | 3467 |
| IGF1R | Insulin like growth factor 1 receptor | 3480 |
| IL10 | Interleukin 10 | 3586 |
| IL12A | Interleukin 12A | 3593 |
| IL12B | Interleukin 12B | 3593 |
| IL12Rb1 | Interleukin 12 receptor subunit beta 1 | 3594 |
| IL13 | Interleukin 13 | 3596 |
| IL15 | Interleukin 15 | 3600 |
| IL16 | Interleukin 16 | 3603 |
| IL17A | Interleukin 17A | 3605 |
| IL17B | Interleukin 17B | 27190 |
| IL17F | Interleukin 17F | 112744 |
| IL17RA | Interleukin 17 receptor A | 23765 |
| IL18 | Interleukin 18(IL18) | 3606 |
| IL18BP | Interleukin-18-binding protein | 10068 |
| IL1A | Interleukin 1 Alpha | 3552 |
| IL1B | Interleukin 1 beta | 3553 |
| IL1F10 | Interleukin 1 family member 10 (theta) | 84639 |
| IL1R1 | Interleukin 1 receptor type 1 | 3554 |
| IL1RL1 | Interleukin 1 receptor like 1 | 9173 |
| IL1RN | Interleukin 1 receptor antagonist | 3557 |
| IL2 | Interleukin 2 | 3558 |
| IL21 | Interleukin 21 | 59067 |
| IL21R | Interleukin 21 receptor | 50615 |
| IL22 | Interleukin 22 | 50616 |
| IL22RA2 | Interleukin 22 Receptor Subunit Alpha 2 | 116379 |
| IL23A | Interleukin 23 subunit alpha | 51561 |
| IL26 | Interleukin 26 | 55801 |
| IL27 | Interleukin 27 | 246778 |
| IL2RA | Interleukin 2 receptor subunit alpha | 3559 |
| IL2RG | Interleukin 2 receptor subunit gamma | 3561 |
| IL3 | Interleukin 3 | 3562 |
| IL33 | Interleukin 33 | 90865 |
| IL36A | Interleukin 36, alpha | 27179 |
| IL36G | Interleukin 36, gamma | 56300 |
| IL36RN | Interleukin 36 receptor antagonist | 26525 |
| IL37 | Interleukin 37 | 27178 |
| IL4 | Interleukin 4 | 3565 |
| IL4R | Interleukin 4 receptor | 3566 |
| IL5 | Interleukin 5 | 3567 |
| IL6 | Interleukin 6 | 3569 |
| IL6R | IL-6 receptor | 3570 |
| IL6ST | Interleukin 6 signal transducer | 3572 |
| IL7R | Interleukin 7 receptor | 3575 |
| IL9 | Interleukin 9 | 3578 |
| IMPDH1 | Inosine monophosphate dehydrogenase 1 | 3614 |
| INS | Insulin | 3630 |
| INSR | Insulin receptor | 3643 |
| IRF3 | Interferon regulatory factor 3 | 3661 |
| IRF5 | Interferon regulatory factor 5 | 3663 |
| IRF7 | Interferon regulatory factor 7 | 3665 |
| ITGAX | CD11c, integrin alpha-X | 3687 |
| ITGB2 | Integrin subunit beta 2 | 3689 |
| KARS | Lysyl-tRNA synthetase | 3735 |
| KDM6B | Llysine demethylase 6B | 23135 |
| KIT | KIT proto-oncogene receptor tyrosine kinase | 3815 |
| KLRC1 | Killer cell lectin like receptor C1 | 3821 |
| KRAS | KRAS Proto-Oncogene, GTPase | 3845 |
| KRT8 | CK8 | 3856 |
| L1CAM | L1 cell adhesion molecule | 3897 |
| LAG3 | Lymphocyte activating 3 | 3902 |
| LAMP1 | Lysosomal associated membrane protein 1 | 3916 |
| LARP1 | La Ribonucleoprotein 1, Translational Regulator | 23367 |
| LCN2 | Lipocalin 2 | 3934 |
| LDHA | Lactate dehydrogenase | 3939 |
| LEPR | Leptin receptor | 3953 |
| LGALS3 | Galectin 3 | 3958 |
| LMNA | Lamin A/C | 4000 |
| LTF | Lactotransferrin | 4057 |
| LYZ | Lysozyme | 4069 |
| LY6E | Lymphocyte Antigen 6 Family Member E | 4061 |
| MAGEA4 | MAGE Family Member A4 | 4103 |
| MAVS | Mitochondrial antiviral signaling protein | 57506 |
| MIF | Macrophage migration inhibitory factor (glycosylation-inhibiting factor) | 4282 |
| MKI67 | Marker Of Proliferation Ki-67 | 4288 |
| MOV10 | Mov10 RISC Complex RNA Helicase | 4343 |
| MPO | Myeloperoxidase | 4353 |
| MRPS7 | Mitochondrial ribosomal protein S7 | 51081 |
| MUC1 | Mucin 1, Cell Surface Associated | 4582 |
| MUC18 | Melanoma cell adhesion molecule | 4162 |
| MVP | Major vault protein | 9961 |
| MX1 | MX dynamin like GTPase 1 | 4599 |
| NCF2 | Neutrophil cytosolic factor 2 | 4688 |
| NCL | Nucleolin | 4691 |
| NCOA6 | Nuclear Receptor Coactivator 6 | 23054 |
| NFKB1 | Nuclear factor kappa B subunit 1 | 4790 |
| NLRP3 | NLR family pyrin domain containing 3 | 114548 |
| NONO | Non-POU domain containing, octamer-binding | 4841 |
| NPM1 | Nucleophosmin | 4869 |
| NPPB | Natriuretic peptide B | 4879 |
| NR4A1 | Nuclear receptor subfamily 4 group A member 1 | 3164 |
| NRBF2 | Nuclear receptor binding factor 2 | 29982 |
| NRP1 | Neuropilin-1 | 8829 |
| NRP2 | Neuropilin-2 | 8828 |
| NUMA1 | Nuclear Mitotic Apparatus Protein 1 | 4926 |
| OAS1 | 2'-5'-oligoadenylate synthetase 1 | 4938 |
| p53 | Tumor Protein P53 | 7157 |
| p62/SQSTM1 | Ubiquitin-Binding Protein P62 | 8878 |
| PCNA | Proliferating cell nuclear antigen | 5111 |
| PDCD1 | Programmed cell death 1 | 5133 |
| PDCD1LG2 | Programmed cell death 1 ligand 2 | 80380 |
| PDGFB | Platelet derived growth factor subunit B | 5155 |
| PDGFD | Platelet derived growth factor D | 80310 |
| PECAM1 | Platelet and endothelial cell adhesion molecule 1 | 5175 |
| PF4 | Platelet Factor 4 | 5196 |
| PLAUR | Plasminogen activator, urokinase receptor | 5329 |
| PLVAP | Plasmalemma vesicle associated protein | 83483 |
| POP1 | POP1 Homolog, Ribonuclease P/MRP Subunit | 10940 |
| POU2AF1 | POU class 2 homeobox associating factor 1 | 5450 |
| POU3F3 | POU class 3 homeobox 3 | 5455 |
| PPL | Periplakin | 5493 |
| PPP1CC | Protein phosphatase 1 catalytic subunit gamma | 5501 |
| PRF1 | Perforin 1 | 5551 |
| PRKRA | Protein activator of interferon induced protein kinase EIF2AK2 | 8575 |
| PRTN3 | Proteinase 3 | 5657 |
| PTPRN | Protein tyrosine phosphatase receptor type N | 5798 |
| PTPRN2 | Protein tyrosine phosphatase, receptor type, N polypeptide 2 | 5799 |
| PVR | Poliovirus receptor CD155 | 5817 |
| RAE1 | Ribonucleic Acid Export 1 | 8480 |
| RALY | RALY heterogeneous nuclear ribonucleoprotein | 22913 |
| RBMS1 | RNA binding motif single stranded interacting protein 1 | 5937 |
| RIPK1 | Receptor interacting serine/threonine kinase 1 | 8737 |
| RIPK3 | Receptor interacting serine/threonine kinase 3 | 11035 |
| RNF41 | Ring finger protein 41 | 10193 |
| ROS1 | ROS Proto-Oncogene 1, Receptor Tyrosine Kinase | 6098 |
| RPLP0 | Ribosomal protein lateral stalk subunit P0 | 6175 |
| RPLP1 | Ribosomal protein lateral stalk subunit P1 | 6176 |
| RPLP2 | Ribosomal protein lateral stalk subunit P2 | 6181 |
| RPP25 | Ribonuclease P/MRP subunit p25 | 54913 |
| RTFDC1 | Replication termination factor 2 domain containing 1 | 51507 |
| SAA1 | Serum Amyloid A1 | 6288 |
| S100A12 | S100 calcium binding protein A12 | 6283 |
| S100A14 | S100 calcium binding protein A14 | 57402 |
| S100A6 | S100 calcium binding protein A6 | 6277 |
| S100A8 | S100 calcium binding protein A8 | 6279 |
| S100A9 | S100 calcium binding protein A9 | 6280 |
| S100B | S100 calcium binding protein B | 6285 |
| SDC1 | Syndecan 1 | 6382 |
| SELL | Selectin L | 6402 |
| SELP | P-selectin | 6403 |
| SERPINB3 | Serpin Family B Member 3 | 6317 |
| SERPINB4 | Serpin Family B Member 4 | 6318 |
| SET | SET Nuclear Proto-Oncogene | 6418 |
| SH3KBP1 | SH3 domain containing kinase binding protein 1 | 30011 |
| SLC30A8 | Solute carrier family 30 member 8 | 169026 |
| SNRNP70 | Small nuclear ribonucleoprotein U1 subunit 70 | 6625 |
| SNRPA | Small Nuclear Ribonucleoprotein Polypeptide A | 6626 |
| SNRPB | Small nuclear ribonucleoprotein polypeptides B and B1 | 6628 |
| SNRPB2 | Small nuclear ribonucleoprotein polypeptide B2 | 6629 |
| SNRPC | Small nuclear ribonucleoprotein polypeptide C | 6631 |
| SNRPD1 | Small nuclear ribonucleoprotein D1 polypeptide | 6632 |
| SNRPD3 | Small nuclear ribonucleoprotein D3 polypeptide | 6634 |
| SNRPN | Small nuclear ribonucleoprotein polypeptide N | 6638 |
| SOX13 | Transcription factor SOX-13 | 9580 |
| SOX2 | SOX2 | 6657 |
| Sp100 | SP100 nuclear antigen | 6672 |
| SPP1 | Secreted phosphoprotein 1 | 6696 |
| SRP19 | Signal Recognition Particle 19 | 6728 |
| SRP54 | Signal recognition particle 54 | 6729 |
| SRSF1 | Serine And Arginine Rich Splicing Factor 1 | 6426 |
| SSB | Sjogren syndrome antigen B | 6741 |
| SSX1 | SSX1 | 6756 |
| SSX2 | SSX family member 2 | 6757 |
| SYK | Tyrosine-proteinkinase SYK | 6850 |
| TAGLN2 | Tansgelin 2 | 8407 |
| TARS | Threonyl-tRNA synthetase | 6897 |
| TBK1 | TANK binding kinase 1 | 29110 |
| TBX21 | IFN-inducible transcription factor T-bet | 3009 |
| TDRD12 | GBU4-5 | 91646 |
| TF | Tansferrin | 7018 |
| TFPI | Tissue Factor Pathway Inhibitor | 7035 |
| TG | Thyroglobulin | 7038 |
| TGFa | Transforming Growth Factor Alpha | 7039 |
| TGFB1 | Transforming growth factor beta 1 | 7040 |
| TGFBR2 | Transforming growth factor beta receptor 2 | 7048 |
| TICAM1 | Toll like receptor adaptor molecule 1 | 148022 |
| TIGIT | T cell immunoreceptor with Ig and ITIM domains | 201633 |
| TLE1 | TLE Family Member 1, Transcriptional Corepressor | 7088 |
| TLR2 | Toll like receptor 2 | 7097 |
| TLR3 | Toll like receptor 3 | 7098 |
| TLR7 | Toll like receptor 7 | 51284 |
| TLR8 | Toll like receptor 8 | 51311 |
| TMIGD2 | Transmembrane and immunoglobulin domain containing 2 | 126259 |
| TMPO | Thymopoietin | 7112 |
| TMPRSS2 | Transmembrane protease, serine 2 | 7113 |
| TNF | Tumour necrosis factor | 7124 |
| TNFRSF10B | TNF receptor superfamily member 10b | 8795 |
| TNFRSF11B | TNF receptor superfamily member 11b | 4982 |
| TNFRSF14 | TNF receptor superfamily member 14 | 8764 |
| TNFRSF18 | TNF receptor superfamily member 18 | 8784 |
| TNFRSF1B | TNF receptor superfamily member 1B | 7133 |
| TNFRSF4 | TNF receptor superfamily member 4 | 7293 |
| TNFRSF8 | TNF receptor superfamily member 8 | 943 |
| TNFRSF9 | TNF receptor superfamily member 9 | 3604 |
| TNFSF13 | Tumor necrosis factor superfamily member 13 | 8741 |
| TNFSF13B | TNF Superfamily Member 13b | 10673 |
| TNFSF14 | Tumor necrosis factor superfamily member 14 | 8740 |
| TOP1 | Topoisomerase (DNA) I | 7150 |
| TOP1MT | Topoisomerase (DNA) I, mitochondrial | 116447 |
| TPO | Thyroid peroxidase | 7173 |
| TRAF3IP3 | TRAF3 interacting protein 3 | 80342 |
| TRIM21 | Tripartite motif containing 21 | 6737 |
| TRIM25 | Tripartite Motif Containing 25 | 7706 |
| TRIM33 | Tripartite motif containing 33 | 51592 |
| TROVE2 | TROVE domain family member 2 | 6738 |
| TSHR | Thyrotropin receptor | 7253 |
| UBA1 | Ubiquitin Activating Enzyme E1/UBA1 Protein, Human, Recombinant | 7317 |
| UBTF | Upstream binding transcription factor, RNA polymerase I | 7343 |
| UNC93B1 | Unc-93 homolog B1, TLR signaling regulator | 81622 |
| VEGFA | Vascular endothelial growth factor A | 7422 |
| VEGFB | Vascular endothelial growth factor B | 7423 |
| VIM | Vimentin | 7431 |
| VSIR | Chromosome 10 open reading frame 54(C10orf54) | 64115 |
| VTCN1 | V-set domain containing T cell activation inhibitor 1 | 79679 |
| vWF | Von Willebrand factor | 7450 |
| XCL1 | X-C motif chemokine ligand 1 | 6375 |
| XRCC5 | X-ray repair cross complementing 5 | 7520 |
| XRCC6 | X-ray repair cross complementing 6 | 2547 |
| YTHDF2 | YTH N6-methyladenosine RNA binding protein 2 | 51441 |
| ZNF574 | Zinc finger protein 574(ZNF574) | 64763 |
| ZYX | Zyxin | 7791 |

**Supplementary Table 3. List of antigens used in viral protein bead arrays.**

| **Symbol** | **Virus** | **Name** |
| --- | --- | --- |
| 229E-N-1:389 | HCoV-229E | Nucleocapsid |
| 229E-S1-16:536 | HCoV-229E | Spike (aa16:536) |
| 229E-S1-S2-ECD-16:1115 | HCoV-229E | Spike ECD (aa16-1115) |
| HKU1-N-1:441 | HCoV-HKU1 | Coronavirus Nucleocapsid |
| HKU1-S1-1:760 | HCoV-HKU1 | (Isolate N1) Spike (aa1:760) |
| HKU1-S1-13:756 | HCoV-HKU1 | (Isolate N5) Spike (aa13-756) |
| HKU1-S1-S2-ECD-1:1295 | HCoV-HKU1 | (Isolate N5) Spike Protein ECD (aa1-1295) |
| IA-H1-B18 | Influenza A | Hemaglutinin |
| IA-H1-GM19 | Influenza A | Hemaglutinin |
| IA-H1-H19 | Influenza A | Hemaglutinin |
| IA-H3-HK2671 | Influenza A | Hemaglutinin |
| IA-H3-HK45 | Influenza A | Hemaglutinin |
| IA-H3-K17 | Influenza A | Hemaglutinin |
| IB-HA-C17 | Influenza B | Hemaglutinin |
| IB-HA-P13 | Influenza B | Hemaglutinin |
| IB-HA-W19 | Influenza B | Hemaglutinin |
| IB-NA-C17 | Influenza B | Neuraminidase |
| MERS-S1-1:1297 | MERS-CoV | Spike ECD (aa 1-1297) |
| MERS-S1-1:725 | MERS-CoV | Spike S1 Subunit (aa1-725) |
| MERS-S2-726:1296 | MERS-CoV | Spike S2 Subunit (aa726-1296) |
| MERS-S-RBD-367:606 | MERS-CoV | Spike RBD (aa 367-606) |
| NL63-N-1:377 | HCoV-NL63 | Nucleocapsid |
| NL63-S1-19:717 | HCoV-NL63 | Spike/S1 Protein (aa19-717) |
| NL63-S1-S2-ECD-1:1296 | HCoV-NL63 | Spike ECD (aa1-1296) |
| OC43-Esterase | HCoV-OC43 | Hemagglutinin Esterase Protein |
| OC43-N-1:448 | HCoV-OC43 | Nucleocapsid |
| OC43-S-1:794 | HCoV-OC43 | Spike (aa1-794) |
| OC43-S1-S2-ECD-1:1304 | HCoV-OC43 | Spike ECD (aa1-1304) |
| OC43-S-766:1304 | HCoV-OC43 | Spike (aa766-1304) |
| SC2_NSP10 | SARS-CoV-2 | NSP1 Protein |
| SC2-3Clpro | SARS-CoV-2 | 3C-like protease |
| SC2-E | SARS-CoV-2 | Envelope protein |
| SC2-H | SARS-CoV-2 | Helicase |
| SC2-M | SARS-CoV-2 | M protein |
| SC2-Mtase | SARS-CoV-2 | Methyltransferase |
| SC2-N | SARS-CoV-2 | N protein |
| SC2-N-1:413 | MERS-CoV-1 | Nucleoprotein |
| SC2-N-1:419 | SARS-CoV-2 | Nucleocapsid |
| SC2-N-1:419-2 | SARS-CoV-2 | Nucleocapsid |
| SC2-N-1:419-3 | SARS-CoV-2 | Nucleocapsid |
| SC2-N-1:419-G335A | SARS-CoV-2 | Nucleocapsid |
| SC2-N-1:422 | SARS-CoV-1 | Nucleoprotein |
| SC2-N-2:419 | SARS-CoV-2 | Nucleocapsid protein |
| SC2-N-2-430 | SARS-CoV-2 | Nucleoprotein (aa1-430) |
| SC2-N7-Mtase | SARS-CoV-2 | N7-Mtase protein |
| SC2-NSP1 | SARS-CoV-2 | NSP10 |
| SC2-NSP10 | SARS-CoV-2 | NSP10 protein |
| SC2-NSP12-1-397 | SARS-CoV-2 | NSP12 protein (aa1-397) |
| SC2-NSP12-398-932 | SARS-CoV-2 | NSP12 protein (aa398-932) |
| SC2-NSP15 | SARS-CoV-2 | NSP15 protein |
| SC2-NSP16 | SARS-CoV-2 | NSP16 protein |
| SC2-NSP2 | SARS-CoV-2 | NSP2 protein |
| SC2-NSP3-1071-1329 | SARS-CoV-2 | NSP3 protein (aa1071-1329) |
| SC2-NSP3-1330-1945 | SARS-CoV-2 | NSP3 protein (aa1330-1945) |
| SC2-NSP3-1-412 | SARS-CoV-2 | NSP3 protein (ss1-412) |
| SC2-NSP3-205:379 | SARS-CoV-2 | NSP3 |
| SC2-NSP3-413-1070 | SARS-CoV-2 | NSP3 protein (aa413-1070) |
| SC2-NSP4 | SARS-CoV-2 | NSP4 protein |
| SC2-NSP5 | SARS-CoV-2 | NSP5 protein |
| SC2-NSP6 | SARS-CoV-2 | NSP6 protein |
| SC2-NSP7 | SARS-CoV-2 | NSP7 Protein |
| SC2-NSP8 | SARS-CoV-2 | NSP8 Protein |
| SC2-NSP9 | SARS-CoV-2 | NSP9 protein |
| SC2-ORF14 | SARS-CoV-2 | ORF14 protein |
| SC2-ORF3a | SARS-CoV-2 | ORF3a protein |
| SC2-ORF7a | SARS-CoV-2 | ORF7a protein |
| SC2-ORF8 | SARS-CoV-2 | ORF8 protein |
| SC2-ORF9b | SARS-CoV-2 | ORF9b protein |
| SC2-Plpro-1541:1859 | SARS-CoV-2 | Papain-like protease |
| SC2-Plpro-1564:1880 | SARS-CoV-2 | Papain-like protease (aa 1564-1880) |
| SC2-S1 | SARS-CoV-2 | Spike S1 |
| SC2-S1+S2-B.1.351 | SARS-CoV-2 | Spike S1+S2 (L18F, D80A, D215G, LAL242-244 deletion, R246I, K417N, E484K, N501Y, D614G, A701V) |
| SC2-S1-1:667 | SARS-CoV-1 | Spike (aa1:667) |
| SC2-S1-11:683 | SARS-CoV-2 | Spike (aa11-683) |
| SC2-S1-13:606 | SARS-CoV-2 | Spike (aa13-606) |
| SC2-S1-16:675 | SARS-CoV-2 | Spike (aa16-675) |
| SC2-S1-16:685 | SARS-CoV-2 | Spike (aa16-685) |
| SC2-S1-16:685-D614G | SARS-CoV-2 | Spike S1 clade G (D614G, aa16-685) |
| SC2-S1-B.1.429 | SARS-CoV-2 | Spike S1 (W152C, L452R, D614G) |
| SC2-S1-B.1.617 | SARS-CoV-2 | Spike (E154K, L452R, E484Q, D614G, P681R) |
| SC2-S1-S2-14:1211 | SARS-CoV-2 | Spike (aa14-1211) |
| SC2-S1-S2-16:1213 | SARS-CoV-2 | Spike (clade G, aa16-1213) |
| SC2-S1-S2-B.1.1.7 | SARS-CoV-2 | Spike B1.1.7 mutation |
| SC2-S1-S2-B.1.351 | SARS-CoV-2 | Spike B.1.351 mutation |
| SC2-S1-S2-ECD-1:1213 | SARS-CoV-2 | Spike ECD (aa1:1213) |
| SC2-S1-S2-ECD-16:1213 | SARS-CoV-2 | Spike S1+S2 ECD (aa16:1213) |
| SC2-S1-S2-P.1 | SARS-CoV-2 | Spike S1+S2 (L18F, T20N, P26S, D138Y, R190S, K417T, E484K, N501Y, D614G, H655Y, T1027I, V1176F) |
| SC2-S2-1 | SARS-CoV-2 | S2 clone 1 |
| SC2-S2-2 | SARS-CoV-2 | S2 clone 2 |
| SC2-S2-ECD-686:1213 | SARS-CoV-2 | Spike ECD (aa686-1213) |
| SC2-S-ECD-16:1213 | SARS-CoV-2 | Spike ECD (aa16-1213) |
| SC2-S-ECD-B.1.1.7 | SARS-CoV-2 | Spike ECD (HV69-70 deletion, Y144 deletion, N501Y, A570D, D614G, P681H, T716I, S982A, D1118H) |
| SC2-S-RBD | SARS-CoV-2 | Spike RBD |
| SC2-S-RBD-306:527 | SARS-CoV-1 | Spike RBD (aa306:527) |
| SC2-S-RBD-319-541 | SARS-CoV-2 | Spike RBD (aa319-541) |
| SC2-S-RBD-330:530 | SARS-CoV-2 | Spike RBD (aa330-530) |
| SC2-S-RBD-A344S | SARS-CoV-2 | Spike RBD (A344S) |
| SC2-S-RBD-D405V-Q414A | SARS-CoV-2 | Spike RBD (D405V-Q414A) |
| SC2-S-RBD-N354D | SARS-CoV-2 | Spike RBD(N354D) |
| SC2-S-RBD-N354D-D364Y | SARS-CoV-2 | Spike RBD (N354D, D364Y) |
| SC2-S-RBD-N439K | SARS-CoV-2 | Spike RBD (N439K) |
| SC2-S-RBD-N501Y | SARS-CoV-2 | Spike RBD (N501Y) |
| SC2-S-RBD-R408I | SARS-CoV-2 | Spike RBD (R408I) |
| SC2-S-RBD-S477N | SARS-CoV-2 | Spike RBD (S477N) |
| SC2-S-RBD-V367F | SARS-CoV-2 | Spike RBD (V367F) |
| SC2-S-RBD-V483A | SARS-CoV-2 | Spike RBD (V483A) |
| SC2-S-RBD-V483I | SARS-CoV-2 | Spike RBD (V483I) |
| SC2-S-RBD-W436R | SARS-CoV-2 | Spike RBD (W436R) |
| SC2-S-RBD-Y453F | SARS-CoV-2 | Spike RBD (Y453F) |
| SC-S1-S2-ECD-1:1195 | SARS-CoV-1 | Spike ECD (S577A, Isolate Tor2, aa1:1195) |

**Supplementary Table 4. Summary of array proteins for antibody and autoantibody detection.**

| **Summary of stock** | **Human targets** | **Viral targets** |
| --- | --- | --- |
| Total | 441 | 114 |
| **Summary of categories** |  |  |
| Autoimmunity panel | 103 |  |
| Immune response panel | 139 |  |
| Pulmonary panel | 16 |  |
| Ig controls | 3 |  |
| Neutralizing antibodies / therapeutics | 6 | 31 |
| Diagnostic / gold standard | 22 | 77 |
| Cross reactivity / specificity | 2 | 35 |
| Immunogenic Epitopes | 6 | 85 |
| Autoimmunity and COVID-19 | 1 |  |
| Disease severity | 18 | 38 |
| Discovery / other | 114 | 52 |
| **Virus species** |  | **Targets** |
| MERS-CoV |  | 5 |
| SARS-CoV |  | 5 |
| HCoV-HKU1 |  | 4 |
| HCoV-NL63 |  | 3 |
| HCoV-OC43 |  | 5 |
| HCoV-229E |  | 3 |
| SARS-CoV-2 |  | 79 |
| Influenza A |  | 6 |
| Influenza B |  | 4 |

**Supplementary Table 5. List of peptides used in viral peptide bead array.**

| **Peptide name** | **Virus** | **Domain** | **Sequence start** | **Sequence end** |
| --- | --- | --- | --- | --- |
| SC2-S_P.1011-1028 | SARS-CoV-2 | Spike CH aa 984-1035 | 1011 | 1028 |
| SC2-S_P.101-118 | SARS-CoV-2 | Spike NTD aa13-305 | 101 | 118 |
| SC2-S_P.1058-1068 | SARS-CoV-2 | Spike CH aa 984-1035 | 1058 | 1068 |
| SC2-S_P.1101-1115 | SARS-CoV-2 | Spike CD aa 1076-1141 | 1101 | 1115 |
| SC2-S_P.1138-1161 | SARS-CoV-2 | Spike CD aa 1076-1141 | 1138 | 1161 |
| SC2-S_P.1144-1165 | SARS-CoV-2 | Spike S2 aa 686-1273 | 1144 | 1165 |
| SC2-S_P.1146-1165 | SARS-CoV-2 | Spike S2 aa 686-1273 | 1146 | 1165 |
| SC2-S_P.1147-1158 | SARS-CoV-2 | Spike S2 aa 686-1273 | 1147 | 1158 |
| SC2-S_P.1161-1177 | SARS-CoV-2 | Spike S2 aa 686-1273 | 1161 | 1177 |
| SC2-S_P.1185-1193 | SARS-CoV-2 | Spike HR2 aa 1163-1213 | 1185 | 1193 |
| SC2-S_P.1192-1200 | SARS-CoV-2 | Spike HR2 aa 1163-1213 | 1192 | 1200 |
| SC2-S_P.1208-1216 | SARS-CoV-2 | Spike HR2 aa 1163-1213 | 1208 | 1216 |
| SC2-S_P.1256-1270 | SARS-CoV-2 | Spike CT aa 1237-1273 | 1256 | 1270 |
| SC2-S_P.16-30 | SARS-CoV-2 | Spike NTD aa13-305 | 16 | 30 |
| SC2-S_P.167-178 | SARS-CoV-2 | Spike NTD aa13-305 | 167 | 178 |
| SC2-S_P.173-185 | SARS-CoV-2 | Spike NTD aa13-305 | 173 | 185 |
| SC2-S_P.210-223 | SARS-CoV-2 | Spike NTD aa13-305 | 210 | 223 |
| SC2-S_P.210-226 | SARS-CoV-2 | Spike NTD aa13-305 | 210 | 226 |
| SC2-S_P.21-45 | SARS-CoV-2 | Spike NTD aa13-305 | 21 | 45 |
| SC2-S_P.221-245 | SARS-CoV-2 | Spike NTD aa13-305 | 221 | 245 |
| SC2-S_P.235-249 | SARS-CoV-2 | Spike NTD aa13-305 | 235 | 249 |
| SC2-S_P.236-258 | SARS-CoV-2 | Spike NTD aa13-305 | 236 | 258 |
| SC2-S_P.261-285 | SARS-CoV-2 | Spike NTD aa13-305 | 261 | 285 |
| SC2-S_P.287-317 | SARS-CoV-2 | Spike NTD aa13-305 | 287 | 317 |
| SC2-S_P.304-321 | SARS-CoV-2 | Spike RBD aa 319-541 | 304 | 321 |
| SC2-S_P.331-349 | SARS-CoV-2 | Spike RBD aa 319-541 | 331 | 349 |
| SC2-S_P.371-387 | SARS-CoV-2 | Spike RBD aa 319-541 | 371 | 387 |
| SC2-S_P.373-390 | SARS-CoV-2 | Spike RBD aa 319-541 | 373 | 390 |
| SC2-S_P.375-394 | SARS-CoV-2 | Spike RBD aa 319-541 | 375 | 394 |
| SC2-S_P.424-433 | SARS-CoV-2 | Spike RBD aa 319-541 | 424 | 433 |
| SC2-S_P.429-448 | SARS-CoV-2 | Spike RBM aa 437-508 | 429 | 448 |
| SC2-S_P.440-457 | SARS-CoV-2 | Spike RBM aa 437-508 | 440 | 457 |
| SC2-S_P.450-469 | SARS-CoV-2 | Spike RBD aa 319-541 | 450 | 469 |
| SC2-S_P.451-468 | SARS-CoV-2 | Spike RBD aa 319-541 | 451 | 468 |
| SC2-S_P.462-474 | SARS-CoV-2 | Spike RBD aa 319-541 | 462 | 474 |
| SC2-S_P.467-487 | SARS-CoV-2 | Spike RBD aa 319-541 | 467 | 487 |
| SC2-S_P.480-499 | SARS-CoV-2 | Spike RBD aa 319-541 | 480 | 499 |
| SC2-S_P.489-506 | SARS-CoV-2 | Spike RBD aa 319-541 | 489 | 506 |
| SC2-S_P.522-546 | SARS-CoV-2 | Spike RBD aa 319-541 | 522 | 546 |
| SC2-S_P.547-570 | SARS-CoV-2 | Spike SD1-SD2 aa 541-685 | 547 | 570 |
| SC2-S_P.551-568 | SARS-CoV-2 | Spike SD1-SD2 aa 541-685 | 551 | 568 |
| SC2-S_P.553-570 | SARS-CoV-2 | Spike SD1-SD2 aa 541-685 | 553 | 570 |
| SC2-S_P.553-571 | SARS-CoV-2 | Spike SD1-SD2 aa 541-685 | 553 | 571 |
| SC2-S_P.554-573 | SARS-CoV-2 | Spike SD1-SD2 aa 541-685 | 554 | 573 |
| SC2-S_P.573-588 | SARS-CoV-2 | Spike SD1-SD2 aa 541-685 | 573 | 588 |
| SC2-S_P.574-593 | SARS-CoV-2 | Spike SD1-SD2 aa 541-685 | 574 | 593 |
| SC2-S_P.607-633 | SARS-CoV-2 | Spike SD1-SD2 aa 541-685 | 607 | 633 |
| SC2-S_P.61-76 | SARS-CoV-2 | Spike NTD aa13-305 | 61 | 76 |
| SC2-S_P.654-673 | SARS-CoV-2 | Spike SD1-SD2 aa 541-685 | 654 | 673 |
| SC2-S_P.655-670 | SARS-CoV-2 | Spike SD1-SD2 aa 541-685 | 655 | 670 |
| SC2-S_P.666-676 | SARS-CoV-2 | Spike SD1-SD2 aa 541-685 | 666 | 676 |
| SC2-S_P.672-685 | SARS-CoV-2 | Spike Furin aa 685-686 | 672 | 685 |
| SC2-S_P.672-687 | SARS-CoV-2 | Spike Furin aa 685-686 | 672 | 687 |
| SC2-S_P.684-706 | SARS-CoV-2 | Spike Furin aa 685-686 | 684 | 706 |
| SC2-S_P.690-707 | SARS-CoV-2 | Spike S2 aa 686-1273 | 690 | 707 |
| SC2-S_P.691-699 | SARS-CoV-2 | Spike S2 aa 686-1273 | 691 | 699 |
| SC2-S_P.747-763 | SARS-CoV-2 | Spike S2 aa 686-1273 | 747 | 763 |
| SC2-S_P.765-785 | SARS-CoV-2 | Spike S2 aa 686-1273 | 765 | 785 |
| SC2-S_P.769-786 | SARS-CoV-2 | Spike S2 aa 686-1273 | 769 | 786 |
| SC2-S_P.771-787 | SARS-CoV-2 | Spike S2 aa 686-1273 | 771 | 787 |
| SC2-S_P.782-802 | SARS-CoV-2 | Spike FP aa 788-806 | 782 | 802 |
| SC2-S_P.782-805 | SARS-CoV-2 | Spike FP aa 788-806 | 782 | 805 |
| SC2-S_P.791-808 | SARS-CoV-2 | Spike S2 aa 686-1273 | 791 | 808 |
| SC2-S_P.802-819 | SARS-CoV-2 | Spike TMPRSS2 aa 815-816 | 802 | 819 |
| SC2-S_P.803-816 | SARS-CoV-2 | Spike TMPRSS2 aa 815-816 | 803 | 816 |
| SC2-S_P.806-820 | SARS-CoV-2 | Spike TMPRSS2 aa 815-816 | 806 | 820 |
| SC2-S_P.806-825 | SARS-CoV-2 | Spike TMPRSS2 aa 815-816 | 806 | 825 |
| SC2-S_P.808-830 | SARS-CoV-2 | Spike TMPRSS2 aa 815-816 | 808 | 830 |
| SC2-S_P.810-826 | SARS-CoV-2 | Spike TMPRSS2 aa 815-816 | 810 | 826 |
| SC2-S_P.811-828 | SARS-CoV-2 | Spike TMPRSS2 aa 815-816 | 811 | 828 |
| SC2-S_P.814-827 | SARS-CoV-2 | Spike TMPRSS2 aa 815-816 | 814 | 827 |
| SC2-S_P.855-869 | SARS-CoV-2 | Spike S2 aa 686-1273 | 855 | 869 |
| SC2-S_P.865-873 | SARS-CoV-2 | Spike S2 aa 686-1273 | 865 | 873 |
| SC2-S_P.902-926 | SARS-CoV-2 | Spike HR1 aa 912-984 | 902 | 926 |
| SC2-S_P.936-952 | SARS-CoV-2 | Spike HR1 aa 912-984 | 936 | 952 |
| SC2-S_P.958-966 | SARS-CoV-2 | Spike HR1 aa 912-984 | 958 | 966 |
| SC2-S_P.976-984 | SARS-CoV-2 | Spike HR1 aa 912-984 | 976 | 984 |
| SC2-S_P.996-1004 | SARS-CoV-2 | Spike CH aa 984-1035 | 996 | 1004 |
| SC2-ORF3_P.112-120 | SARS-CoV-2 | ORF3 | 112 | 120 |
| SC2-ORF3_P.211-219 | SARS-CoV-2 | ORF3 | 211 | 219 |
| SC2-ORF3_P.4-18 | SARS-CoV-2 | ORF3 | 4 | 18 |
| SC2-ORF3_P.72-80 | SARS-CoV-2 | ORF3 | 72 | 80 |
| SC2-ORF3a_P.12-20 | SARS-CoV-2 | ORF3 | 12 | 20 |
| SC2-ORF3a_P.181-203 | SARS-CoV-2 | ORF3 | 181 | 203 |
| SC2-ORF3a_P.6-20 | SARS-CoV-2 | ORF3 | 6 | 20 |
| SC2-E_P.52-66 | SARS-CoV-2 | E protein intravirion aa 35-75 | 52 | 66 |
| SC2-E_P.56-65 | SARS-CoV-2 | E protein intravirion aa 35-75 | 56 | 65 |
| SC2-E_P.56-70 | SARS-CoV-2 | E protein intravirion aa 35-75 | 56 | 70 |
| SC2-M_P.1-19 | SARS-CoV-2 | M protein virion surface aa 2-19 | 1 | 19 |
| SC2-M_P.1-24 | SARS-CoV-2 | M protein virion surface aa 2-19 | 1 | 24 |
| SC2-M_P.133-151 | SARS-CoV-2 | M protein intravirion aa 101-222 | 133 | 151 |
| SC2-M_P.136-145 | SARS-CoV-2 | M protein intravirion aa 101-222 | 136 | 145 |
| SC2-M_P.148-156 | SARS-CoV-2 | M protein intravirion aa 101-222 | 148 | 156 |
| SC2-M_P.160-173 | SARS-CoV-2 | M protein intravirion aa 101-222 | 160 | 173 |
| SC2-M_P.176-190 | SARS-CoV-2 | M protein intravirion aa 101-222 | 176 | 190 |
| SC2-M_P.185-199 | SARS-CoV-2 | M protein intravirion aa 101-222 | 185 | 199 |
| SC2-M_P.43-51 | SARS-CoV-2 | M protein intravirion aa 41-50 | 43 | 51 |
| SC2-M_P.61-70 | SARS-CoV-2 | M protein helical aa 51-71 | 61 | 70 |
| SC2-M_P.89-97 | SARS-CoV-2 | M protein helical aa 80-100 | 89 | 97 |
| SC2-M_P.97-111 | SARS-CoV-2 | M protein intravirion aa 101-222 | 97 | 111 |
| SC2-ORF6_P.20-31 | SARS-CoV-2 | ORF6 | 20 | 31 |
| SC2-ORF6_P.26-40 | SARS-CoV-2 | ORF6 | 26 | 40 |
| SC2-ORF7_P.40-49 | SARS-CoV-2 | ORF7 | 40 | 49 |
| SC2-ORF7_P.76-85 | SARS-CoV-2 | ORF7 | 76 | 85 |
| SC2-ORF7_P.90-104 | SARS-CoV-2 | ORF7 | 90 | 104 |
| SC2-N_P.107-121 | SARS-CoV-2 | N protein NTD aa 49-175 | 107 | 121 |
| SC2-N_P.1-18 | SARS-CoV-2 | N protein tail aa 1-49 | 1 | 18 |
| SC2-N_P.127-141 | SARS-CoV-2 | N protein NTD aa 49-175 | 127 | 141 |
| SC2-N_P.134-143 | SARS-CoV-2 | N protein NTD aa 49-175 | 134 | 143 |
| SC2-N_P.138-146 | SARS-CoV-2 | N protein NTD aa 49-175 | 138 | 146 |
| SC2-N_P.150-159 | SARS-CoV-2 | N protein NTD aa 49-175 | 150 | 159 |
| SC2-N_P.152-172 | SARS-CoV-2 | N protein NTD aa 49-175 | 152 | 172 |
| SC2-N_P.153-169 | SARS-CoV-2 | N protein NTD aa 49-175 | 153 | 169 |
| SC2-N_P.153-171 | SARS-CoV-2 | N protein linker aa 175-247 | 153 | 171 |
| SC2-N_P.153-172 | SARS-CoV-2 | N protein NTD aa 49-175 | 153 | 172 |
| SC2-N_P.153-176 | SARS-CoV-2 | N protein NTD aa 49-175 | 153 | 176 |
| SC2-N_P.156-170 | SARS-CoV-2 | N protein linker aa 175-247 | 156 | 170 |
| SC2-N_P.159-167 | SARS-CoV-2 | N protein linker aa 175-247 | 159 | 167 |
| SC2-N_P.16-19 | SARS-CoV-2 | N protein tail aa 1-49 | 16 | 19 |
| SC2-N_P.359-375 | SARS-CoV-2 | N protein C-tail aa 365-419 | 359 | 375 |
| SC2-N_P.215-224 | SARS-CoV-2 | N protein linker aa 175-247 | 215 | 224 |
| SC2-N_P.219-227 | SARS-CoV-2 | N protein linker aa 175-247 | 219 | 227 |
| SC2-N_P.221-230 | SARS-CoV-2 | N protein linker aa 175-247 | 221 | 230 |
| SC2-N_P.221-235 | SARS-CoV-2 | N protein linker aa 175-247 | 221 | 235 |
| SC2-N_P.221-244 | SARS-CoV-2 | N protein linker aa 175-247 | 221 | 244 |
| SC2-N_P.222-230 | SARS-CoV-2 | N protein linker aa 175-247 | 222 | 230 |
| SC2-N_P.226-234 | SARS-CoV-2 | N protein linker aa 175-247 | 226 | 234 |
| SC2-N_P.228-251 | SARS-CoV-2 | N protein linker aa 175-247 | 228 | 251 |
| SC2-N_P.245-260 | SARS-CoV-2 | N protein CTD aa 247-365 | 245 | 260 |
| SC2-N_P.245-261 | SARS-CoV-2 | N protein CTD aa 247-365 | 245 | 261 |
| SC2-N_P.264-278 | SARS-CoV-2 | N protein CTD aa 247-365 | 264 | 278 |
| SC2-N_P.265-274 | SARS-CoV-2 | N protein CTD aa 247-365 | 265 | 274 |
| SC2-N_P.277-292 | SARS-CoV-2 | N protein CTD aa 247-365 | 277 | 292 |
| SC2-N_P.297-307 | SARS-CoV-2 | N protein CTD aa 247-365 | 297 | 307 |
| SC2-N_P.298-312 | SARS-CoV-2 | N protein CTD aa 247-365 | 298 | 312 |
| SC2-N_P.311-325 | SARS-CoV-2 | N protein CTD aa 247-365 | 311 | 325 |
| SC2-N_P.316-324 | SARS-CoV-2 | N protein CTD aa 247-365 | 316 | 324 |
| SC2-N_P.322-331 | SARS-CoV-2 | N protein CTD aa 247-365 | 322 | 331 |
| SC2-N_P.328-342 | SARS-CoV-2 | N protein CTD aa 247-365 | 328 | 342 |
| SC2-N_P.345-353 | SARS-CoV-2 | N protein CTD aa 247-365 | 345 | 353 |
| SC2-N_P.358-381 | SARS-CoV-2 | N protein C-tail aa 365-419 | 358 | 381 |
| SC2-N_P.376-385 | SARS-CoV-2 | N protein C-tail aa 365-419 | 376 | 385 |
| SC2-N_P.376-400 | SARS-CoV-2 | N protein C-tail aa 365-419 | 376 | 400 |
| SC2-N_P.393-416 | SARS-CoV-2 | N protein C-tail aa 365-419 | 393 | 416 |
| SC2-N_P.397-411 | SARS-CoV-2 | N protein C-tail aa 365-419 | 397 | 411 |
| SC2-N_P.41-61 | SARS-CoV-2 | N protein tail aa 1-49 | 41 | 61 |
| SC2-N_P.50-64 | SARS-CoV-2 | N protein NTD aa 49-175 | 50 | 64 |
| SC2-N_P.66-74 | SARS-CoV-2 | N protein NTD aa 49-175 | 66 | 74 |
| SC2-N_P.84-98 | SARS-CoV-2 | N protein NTD aa 49-175 | 84 | 98 |
| SC2-N_P.9-17 | SARS-CoV-2 | N protein tail aa 1-49 | 9 | 17 |
| SC2-S_P.150-159 | SARS-CoV-2 | Spike NTD aa13-305 | 150 | 159 |
| SC2-ORF10_P.4-18 | SARS-CoV-2 | ORF10 | 4 | 18 |
| SC2-ORF8_P.39-60 | SARS-CoV-2 | ORF7a | 39 | 60 |
| SC2-ORF8_P.109-118 | SARS-CoV-2 | ORF8 | 109 | 118 |
| SC2-ORF8_P.43-57 | SARS-CoV-2 | ORF8 | 43 | 57 |
| SC2-ORF8_P.66-86 | SARS-CoV-2 | ORF8 | 66 | 86 |
| SC2-ORF1_P.2192-2200 | SARS-CoV-2 | ORF1 | 2192 | 2200 |
| SC2-ORF1_P.294-303 | SARS-CoV-2 | ORF1 | 294 | 303 |
| SC2-ORF1_P.3628-3636 | SARS-CoV-2 | ORF1 | 3628 | 3636 |
| SC2-ORF1_P.3906-3914 | SARS-CoV-2 | ORF1 | 3906 | 3914 |
| SC2-ORF1_P.397-405 | SARS-CoV-2 | ORF1 | 397 | 405 |
| SC2-ORF1_P.5455-5463 | SARS-CoV-2 | ORF1 | 5455 | 5463 |
| SC2-ORF1_P.6751-6765 | SARS-CoV-2 | ORF1 | 6751 | 6765 |
| SC2-ORF1_P.819-828 | SARS-CoV-2 | ORF1 | 819 | 828 |
| SC2-ORF1_P.91-99 | SARS-CoV-2 | ORF1 | 91 | 99 |
| SC2-ORF1_P.98-106 | SARS-CoV-2 | ORF1 | 98 | 106 |
| SC2-ORF1ab_P.142-165 | SARS-CoV-2 | ORF1 | 142 | 165 |
| SC2-ORF1ab_P.1484-1496 | SARS-CoV-2 | ORF1 | 1484 | 1496 |
| SC2-ORF1ab_P.1914-1928 | SARS-CoV-2 | ORF1 | 1914 | 1928 |
| SC2-ORF1ab_P.2211-2218 | SARS-CoV-2 | ORF1 | 2211 | 2218 |
| SC2-ORF1ab_P.2569-2577 | SARS-CoV-2 | ORF1 | 2569 | 2577 |
| SC2-ORF1ab_P.2911-2918 | SARS-CoV-2 | ORF1 | 2911 | 2918 |
| SC2-ORF1ab_P.3484-3499 | SARS-CoV-2 | ORF1 | 3484 | 3499 |
| SC2-ORF1ab_P.3826-3843 | SARS-CoV-2 | ORF1 | 3826 | 3843 |
| SC2-ORF1ab_P.386-400 | SARS-CoV-2 | ORF1 | 386 | 400 |
| SC2-ORF1ab_P.6292-6306 | SARS-CoV-2 | ORF1 | 6292 | 6306 |
| SC2-ORF1ab_P.818-832 | SARS-CoV-2 | ORF1 | 818 | 832 |
| SC2-ORF1ab_P.95-111 | SARS-CoV-2 | ORF1 | 95 | 111 |
| MHV.S_P.700-717 | MHV | Spike | 700 | 717 |
| HIV.gag-pol_P.959-983 | HIV-1 | HIV public epitope | 959 | 983 |
| 229E-S_P.550-567 | HCoV-229E | Spike | 550 | 567 |
| BCoV-S_P.751-758 | BCoV-ENT | Spike | 751 | 758 |
| NL63.S_P.731-748 | HCoV-NL63 | Spike | 731 | 748 |
| NL63.N_P.119-136 | HCoV-NL63 | Nucleocapsid | 119 | 136 |
| HKU1.N_P.166-186 | HCoV-HKU1 | Nucleocapsid | 166 | 186 |
| HHV4-EBNA_P | Human Herpesvirus 4 | EBNA-1 public epitope |  |  |
| MERS-N_P.141-161 | MERS-CoV | Nucleocapsid | 141 | 161 |
| HRV-D1P36_gp1_P.571-594 | HRV-A1 | Glycoprotein 1 | 571 | 594 |
| OC43-S_P.741-758 | HCoV-OC43 | Spike | 741 | 758 |

**Supplementary Table 6. Summary of array peptides for antibody detection.**

| **Virus species** | | **Targets**  **(out of total 192)** |
| --- | --- | --- |
| SARS-CoV-2 | | 178 |
| Other / cross reactivity / control | | 14 |
| **Structural vs non-structural** | | |
| HCoVs structural proteins | Membrane protein | 13 |
|  | Nucleocapsid protein | 47 |
|  | Spike / Surface glycoprotein | 87 |
| HCoVs non-structural proteins and other viral proteins | | 45 |
| **Categories investigated** | | |
| Total exceeds 192 as some are indicated in more than one category | Neutralizing antibodies/ therapeutics | 20 |
|  | Diagnostic use | 30 |
|  | Cross reactivity, specificity | 61 |
|  | Immunogenic epitope | 113 |
|  | Disease severity | 62 |

**Supplementary Table 7. Cohort summary for hospitalized versus non-hospitalized participants.**

| **Characteristics** | **Hospitalized** | **Non-hospitalized** |
| --- | --- | --- |
| Age, mean (SD) | 42.4 (14.2) | 50.5 (15.6) |
| Female, % | 71.9 | 55.9 |
| Race |  |  |
| Black or African American, % | 21.9 | 37.3 |
| White, % | 46.9 | 41.2 |
| Other, % | 20.3 | 11.3 |
| Hispanic, % | 28.1 | 37.7 |
| Days from the start of COVID-19 to sample collection, mean (SD) | 2.9 (2.1) | 3.1 (4.2) |
| Tobacco use, % | 32.8 | 26.0 |
| WHO score, mean (SD) | 2 (0) | 3.7 (0.7) |

**Supplementary Table 8. Cohort summary for hospitalized PASC versus hospitalized non-PASC participants.**

| **Characteristics** | **Hospitalized non-PASC** | **Hospitalized PASC** |
| --- | --- | --- |
| Age, mean (SD) | 47.6 (16.0) | 54.1 (14.2) |
| Female, % | 57.4 | 53.5 |
| Race |  |  |
| Black or African American, % | 39.6 | 36.6 |
| White, % | 40.6 | 39.4 |
| Other, % | 9.4 | 16.9 |
| Hispanic, % | 41.5 | 33.8 |
| Days from the start of COVID-19 to sample collection, mean (SD) | 2.3 (2.6) | 4.1 (5.5) |
| Tobacco use, % | 17.9 | 38.0 |
| WHO score, mean (SD) | 3.6 (0.6) | 3.8 (0.7) |
